# Shear‐Induced Anisotropic Supramolecular Gel Noodles for Improved Cell Guidance in Polarized Tissue Engineering

**DOI:** 10.1002/smll.202513952

**Published:** 2026-03-12

**Authors:** Dipankar Ghosh, Matthew Walker, Lauren Matthews, Charlie Patterson, Oana Dobre, Massimo Vassalli, Dave J. Adams

**Affiliations:** ^1^ School of Chemistry University of Glasgow Glasgow UK; ^2^ Centre For the Cellular Microenvironment University of Glasgow Glasgow UK; ^3^ ISIS Pulsed Neutron and Muon Source Harwell Science and Innovation Campus Didcot UK; ^4^ School of Engineering Rankine Building University of Glasgow Glasgow UK

**Keywords:** 2D SANS, anisotropic alignment, gel noodles, muscle cell alignment, tensile strength

## Abstract

Tissue engineering and regenerative medicine applications require the integration of multifunctional materials, offering tissue‐specific mechanical cues and geometric guidance to support cell differentiation. In this context, supramolecular gel noodles formed by ionic cross‐linking of pre‐assembled micellar networks offer unique opportunities for creating anisotropic soft materials. However, controlling fibrillar alignment over extended lengths remains a challenge. Herein, a shear‐induced method to fabricate 1D gel noodles with enhanced macroscopic alignment using dipeptide‐based gelators is reported. By implementing a two‐stage extrusion protocol, we generate a thin tail segment with a distinct flow history that exhibits higher retained alignment than the pump‐driven segment. Polarized optical microscopy and small‐angle neutron scattering confirm superior fibrillar orientation in the thin segment, and mechanical testing reveals up to ∼400‐fold increase in nominal stress at failure. The method is effective across multiple gelators, demonstrating its broad applicability for tuning macroscopic alignment in gel noodles. Preliminary C2C12 culture on the thin segment demonstrates improved cell adhesion, elongation, and increased MyoD expression compared to the thick segment. These findings provide a scalable route to introduce anisotropy in supramolecular gel noodles through processing history, and we present cell culture data as proof of compatibility and contact guidance relevant to aligned tissue‐mimetic scaffolds.

## Graphical abstract

A simple two‐stage extrusion aligns supramolecular fibrils into 1D gel noodles. The residual pressure in the viscous pre‐gel drives the formation of a thin filament that is locked by Ca^2+^ cross‐linker into a highly anisotropic segment. The aligned noodles show enhanced tensile strength and guide myoblast elongation along the fiber, mimicking aligned tissue architecture.

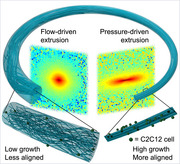

## 1 Introduction

Supramolecular gels, formed by the self‐assembly of low‐molecular‐weight gelators (LMWGs), have attracted significant interest due to their tunable properties, dynamic nature, and biocompatibility [1, 2, 3, 4]. Unlike polymer gels, which are typically robust and retain their shape, supramolecular gels suffer from weak mechanical strength and poor rheological performance, often conforming to the shape of their container [2, 5, 6]. These limitations restrict their potential in applications requiring well‐defined structures and enhanced mechanical properties [5]. Although the supramolecular nature of these gels allows for chemical modifications that tune molecular‐level interactions, such as hydrogen bonding or hydrophilic‐hydrophobic balance, these strategies primarily influence nanoscale assembly and local architecture [7, 8, 9, 10]. Translating such control to the macroscopic scale, however, remains a significant challenge due to the dynamic and reversible nature of supramolecular interactions [11]. Therefore, developing strategies to manipulate the macroscopic structure of these gels without altering the core molecular design remains a central focus of ongoing research [11, 12].

Shaping supramolecular gels into stable, predefined 3D architectures can be achieved through 3D printing, molding, photopatterning, and other fabrication methods [5, 13, 14, 15, 16]. One compelling strategy involves shaping these gels into anisotropic, 1D constructs, commonly referred to as “gel noodles” [17, 18, 19, 20]. These structures offer distinct advantages over conventional bulk gels, including enhanced directional properties and applications in in vivo drug delivery, soft robotics, and tissue engineering [21, 22, 23, 24, 25]. The formation of gel noodles induces alignment within the reinforcing fibrillar network, making them not merely structural novelties but functionally advantageous architectures [12, 26]. Stupp and co‐workers first introduced gel noodles for directed cell growth [17], and later demonstrated that aligned nanofibers within tubular hydrogels can encapsulate and guide the orientation of vascular cells [27]. Building on this foundational work, numerous studies have reported the fabrication of mechanically stable 1D gel noodles [18, 19, 20, 28]. However, many of these constructs exhibit limited internal fibrillar alignment [20, 28]. Moreover, the characterization in existing reports is often limited to microscale observations, typically using polarized light or electron microscopy [22, 29, 30]. Achieving uniform macroscopic alignment across extended gel segments remains challenging, though recent studies have demonstrated promising results in inducing anisotropy in supramolecular gels [12, 24, 31].

Anisotropic alignment can be induced by electric or magnetic fields, shear‐induced extrusion, or directional gelation via dual‐network or hybrid systems [31, 32, 33, 34, 35, 36]. While effective, these approaches often involve complex instrumentation, specialized gelator designs, or limited scalability. For example, magnetic alignment strategies have demonstrated potential, yet they are constrained by the requirement for high‐field magnets and tight temporal control of the gelation process [11, 35, 37]. Extrusion‐based printing of nanofibrillar gels such as cellulose or peptide systems can impart alignment but typically requires precise control over flow dynamics and cross‐linking kinetics [38]. A simple approach to induce macroscopic alignment is to introduce shear‐flow to the gel precursor [36, 39, 40, 41, 42, 43, 44]. For example, a shear‐flow‐based approach reported by Wall et al. aligns supramolecular nanostructures in peptide‐*π*‐conjugated systems for optoelectronic applications [36]; however, aspects such as bulk anisotropy, mechanical robustness, and biocompatibility remain to be explored.

We hypothesize that flow‐induced alignment in the pre‐gel micellar state can be kinetically trapped by Ca^2+^ cross‐linking, enabling a systematic relationship between processing history, retained anisotropy, and function in supramolecular gel noodles. The retained anisotropy or micellar orientation is dictated by the competition between viscoelastic relaxation (extensional relaxation time) and the time scales of deformation and cross‐linking; thus, anisotropy should increase when relaxation is slow relative to processing. Accordingly, we examine how extrusion flow history controls alignment retention in supramolecular gel noodles. We first use a pump‐driven extrusion regime, which we previously used to produce long noodles with consistent geometry [20, 28], followed by a second regime accessed by stopping the syringe pump so that residual pressure in the viscous precursor drives continued flow during pressure relaxation prior to ionic locking. Using this two‐stage ‘pump‐on’ and ‘pump‐off’ extrusion protocol, we test whether distinct flow histories can be encoded within a single noodle to tune retained anisotropy. This method offers a straightforward route to generate anisotropic 1D supramolecular noodles, where tuning the fabrication conditions controls pre‐gel micelle alignment and defines the filament properties. Moreover, aligned supramolecular hydrogels have shown great potential to direct cellular orientation and support tissue‐specific regeneration [45]. For example, peptide amphiphile tubes with aligned nanofibers have been used to guide the alignment of vascular cells [27]. Similarly, aligned gels have helped support nerve repair by promoting the growth of nerve cells along the gel structure [46], and aligned hydrogel channels have been shown to guide axon regrowth after spinal cord injury [47]. Our system builds on these advances by offering a simple method to make strong, aligned gel noodles that could serve as useful scaffolds for growing cells in organized tissues such as muscle or nerve. These materials are also promising for broader applications where directional control and mechanical integrity are essential, including tissue engineering, soft robotics, and flexible electronics [21, 31, 48].

## 2 Results and Discussion

To test the proposed process‐structure‐property link, we quantify precursor viscoelasticity and extensional relaxation as a key processing time scale and then implement the two‐stage extrusion that generates a pump‐driven segment followed by a pressure‐relaxation segment within a single noodle. Previously, we employed syringe‐pump extrusion of dipeptide‐based LMWGs for noodle formation [20, 28]. These dipeptides self‐assemble into long tubular micelles at high pH in aqueous media [49], which can be directionally aligned through external stimuli, including shear forces [50, 51, 52]. We demonstrated that partial alignment could be induced by extruding the pre‐gel solution through a needle, thereby applying shear [20, 53]. Upon extrusion into a Ca^2+^ solution, ionic cross‐linking ‘locks’ this alignment, resulting in gel noodles that exhibit regions of birefringence, indicative of internal fibrillar alignment [53]. However, the degree of alignment was limited and was not quantitatively assessed. Moreover, the mechanical advantages expected from anisotropy were only partially achieved [53]. Hence, we test whether alignment can be controlled by processing history by introducing a pump‐off pressure‐relaxation regime that alters extensional strain accumulation before ionic locking.

**FIGURE 1:**
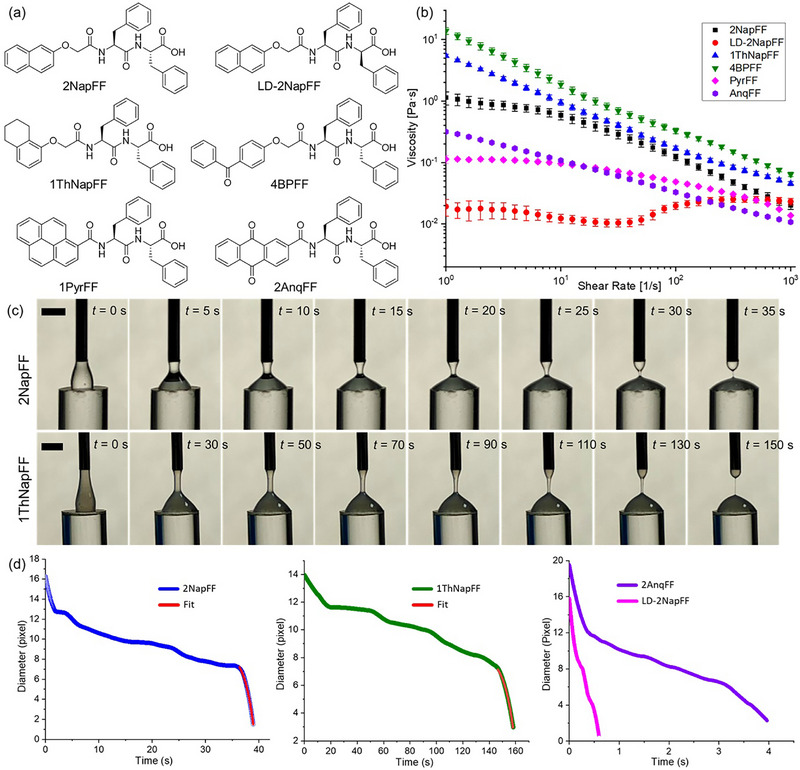
(a) Chemical structures of the dipeptide gelators used in this study. (b) Shear viscosity profiles of the gelator solutions at 40 mg/mL and pH 10.5. The 1PyrFF and 2AnqFF samples were heated at 60°C for 1 h followed by cooling to room temperature; all other samples were used as prepared. (c) Representative images from extensional viscosity experiments showing filament formation and thinning over time during the DoS method for 2NapFF and 1ThNapFF. Scale bars represent 2 mm. (d) Extracted filament diameter vs. time during DoS experiments for 2NapFF, 1ThNapFF, *LD*‐2NapFF, and 2AnqFF. For 2NapFF (blue, left) and 1ThNapFF (green, middle), the elastocapillary tail was fitted to an exponential decay (shown as a red line) to extract 𝜆_𝐸_. For *LD*‐2NapFF and 2AnqFF (right), no clear exponential tail was observed within the recorded window.

A key requirement for forming robust and flexible noodles is the use of a viscous pre‐gel solution containing long cylindrical micelles [54]. When shear is applied, these micelles can align in one direction and be cross‐linked in the presence of a suitable linker. Based on our previous work with dipeptide systems, we selected several diphenylalanine (FF)‐based functionalized dipeptides with different aromatic protecting groups, as they form viscous solutions with cylindrical micellar structures (Figure 1a) [20, 28, 49, 53]. Viscosity measurements were performed at high pH (10.5 ± 0.05, adjusted with NaOH) to determine the optimal concentration. At 40 mg/mL, the solutions were sufficiently viscous to form thin strings (Figure 1b), and this concentration was used for all samples. Since 1PyrFF and 2AnqFF showed relatively lower viscosity, thermal annealing at 60°C for 1 h in closed glass vials was applied to increase viscosity [54, 55]. The resulting heat‐cooled solutions were used in all subsequent studies involving 1PyrFF and 2AnqFF.

To complement the steady shear viscosity data and better understand the response of the solutions under deformation, we measured their extensional viscosity using the dripping‐onto‐substrate (DoS) technique [56]. In this method, a droplet is slowly dispensed from a syringe needle onto a flat glass substrate. As the droplet contacts the surface, a liquid filament forms between the needle tip and substrate. This filament stretches, thins, and eventually breaks due to surface tension. The rate of thinning reflects the balance between capillary forces, which drive the thinning, and elastic forces, which resist it. The filament diameter was tracked over time to extract the extensional relaxation time (*λ_E_*_,_ see Equation 1), the characteristic time over which elastic stresses persist during uniaxial stretching. Higher *λ_E_* values indicate stronger elastic resistance to breakup and are often associated with entangled or aligned micellar networks. Since noodle formation involves extrusion through a narrow needle, the DoS method serves as a preliminary model to assess which solutions are likely to form stable filaments during extrusion. In low‐viscosity solutions, the droplet detached rapidly without forming a stable filament. In contrast, viscoelastic samples formed persistent filaments that thinned gradually, allowing accurate determination of *λ_E_* (Figure 1c). *λ_E_* provides a processing‐relevant time scale for assessing filament persistence during pressure relaxation and for qualitatively rationalizing alignment retention after Ca^2+^ cross‐linking.

The extensional viscosity trends matched well with the solution viscosity profiles. 2NapFF, with a viscosity of ∼1.0 Pa·s and a filament breaking time of ∼40 s (Figure 1d), showed a relaxation time (*λ_E_*) of 0.47 s. 1ThNapFF and 4BPFF exhibited the highest viscosities; 1ThNapFF also formed a very stable filament (breaking time > 150 s) with a longer relaxation time of 2.45 s. For 4BPFF, *λ_E_* could not be determined because the filament remained intact for at least 15 min, and no exponential thinning was observed. In contrast, *LD*‐2NapFF, 1PyrFF, and 2AnqFF showed the lowest viscosities. *LD*‐2NapFF and 2AnqFF both had short breaking times of <5 s (Figure 1d), where a linear decay in filament diameter was observed instead of an exponential one. For 1PyrFF, the droplet fell without forming a stable filament, making it unsuitable for analysis by this method.

However, the solution viscosity or the extensional viscosity solely does not determine the stability or mechanical robustness of the noodle. For example, we previously showed that the tetrabutylammonium (TBA) salt of 2NapFF forms a much more viscous solution with significantly higher extensional viscosity compared to the sodium salt of 2NapFF [57]. Despite this, noodles formed from the TBA salt were fragile and dissolved easily in water due to the presence of branched micelles in the pre‐gel solution [57]. This highlights that specific micellar structure, in addition to viscosity, plays a critical role in determining the mechanical properties of the noodle.

The extensional viscosity profiles indicated that thin strings could be formed from 2NapFF, 1ThNapFF, and 4BPFF solutions. These viscous strings are expected to contain long micellar structures that can align along their length. To test this, we manually ejected the peptide solutions vertically from a syringe. The 2NapFF solution formed long, thin strings (Figure S1) which were immediately immersed into a CaCl_2_ bath by moving the syringe. Upon immersion, the viscous strings gelled into robust filaments due to Ca^2+^ cross‐linking. The resulting filaments showed tensile integrity and could be handled and removed from the bath without deforming. Polarized optical microscopy (POM) images revealed clear fibrillar alignment, much improved compared to traditional gel noodles prepared by syringe pump and spin coater (Figure S2). For 1ThNapFF and 4BPFF, string formation was difficult due to their high viscosities. In contrast, *LD*‐2NapFF, 1PyrFF, and 2AnqFF were too fluid to form stable strings.

**FIGURE 2:**
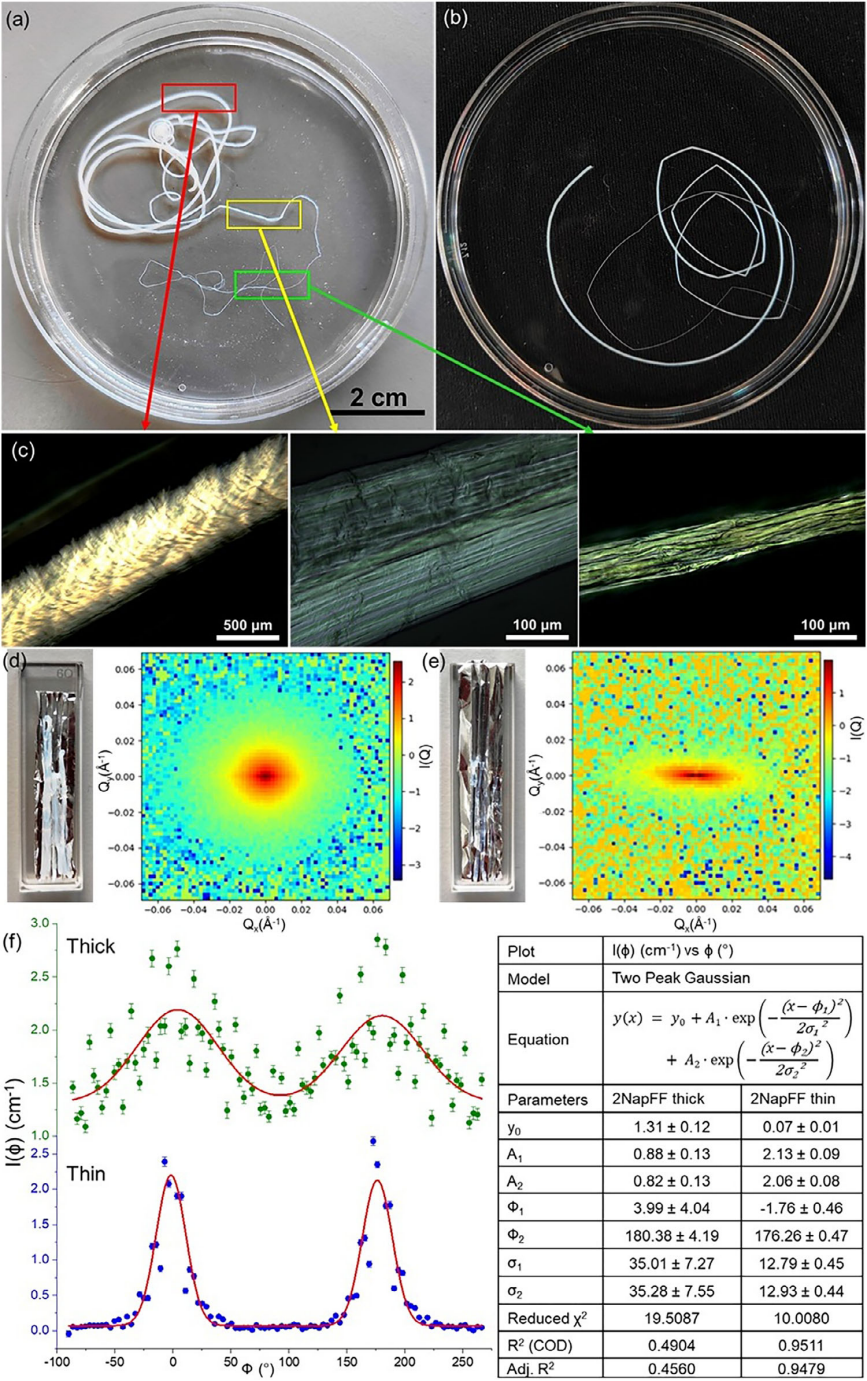
Gel noodles prepared with (a) 2NapFF and (b) *LD*‐2NapFF, showing distinct thick (diameter ∼0.6 mm) and thin (diameter 30–75 µm) segments. In the 2NapFF noodle, the red box highlights the thick segment formed during extrusion using a syringe pump. The yellow box indicates the region formed immediately after the pump was stopped, and the green box shows the extended thin tail that followed. Both petri dishes have a diameter of 90 mm. (c) POM images of the three segments of the 2NapFF noodle corresponding to the red (left), yellow (middle), and green (right) regions in (a), showing improved fibrillar alignment. (d,e) Sample preparation for SANS analysis of the 2NapFF noodle and the corresponding 2D scattering patterns: (d) thick segment, and (e) thin segment showing pronounced anisotropy. (f) Two‐peak Gaussian fitting of the azimuthal intensity extracted from the 2D SANS data of the 2NapFF thick (green) and thin (blue) segments. The corresponding fits are shown as red lines, and the fitting parameters are listed on the right.

To create a controlled and testable difference in flow history within a single extrusion event, we implemented a two‐stage protocol. First, the syringe pump extruded the pre‐gel solution at 3 mL/min for 15–20 s to generate a pump‐on thick segment under high flow [28, 54]. We then stopped the pump and allowed residual pressure in the syringe to relax, producing a pump‐off thin tail segment extruded at a slower rate until pressure decayed. This pump‐off regime introduces a distinct extensional‐flow history and generates a thinner segment (Figure 2a,b; Figure S3). Because both segments originate from the same solution and ionic locking chemistry, differences in alignment and properties can be attributed primarily to processing history rather than composition.

We compared the diameter and fibrillar alignment between the front and tail segments of the gel noodles formed with each gelator. The thicker segment showed birefringence, indicating some degree of internal alignment (Figure 2c). However, the fibrillar structure was not clearly resolved, and individual fibrils were not visible, suggesting that the alignment was less uniform or more dispersed. In contrast, the thinner tail segments displayed clearer birefringence patterns, with well‐aligned fibrils visible along the length (Figure 2c). These thin noodles consistently showed improved alignment in polarized images across all gelators studied (Figures S4–S9). The clear difference indicates that along with chemistry, flow history also is an important control parameter for retained alignment. The pump‐off (thin) regime likely accumulates greater extensional strain before ionic locking, contributing to the higher orientation visible by POM. Importantly, this distinction was observed in all six gelators (Figure 2c; Figures S4–S9), indicating that the approach is broadly applicable. While the improvement of alignment was consistent, the length and diameter of the thin tail segments varied. Since this portion forms due to residual pressure in the syringe after stopping the pump, extrusion typically stops within a few seconds, resulting in tail lengths of 15–30 cm. The thin segments extended longest (∼30 cm) for 2NapFF and 4BPFF, which may be due to their higher extensional viscosity. For 2NapFF, the diameter of the thin segment was as low as ∼30 µm, nearly 20 times thinner than the thick segment. *LD*‐2NapFF and 1ThNapFF also formed thin segments about 10 times thinner, while for 1PyrFF and 2AnqFF, the difference was more modest, with the thin segments being about 3–5 times thinner.

It is important to note that noodle thickness does not directly correlate with fibrillar alignment. For example, extruding 2NapFF through a thinner needle results in thinner noodles but does not improve fibril alignment. When 2NapFF gel noodles were prepared using a 25 G needle, the resulting noodles (Figure S10) were fragile and exhibited low tensile integrity, breaking at lengths of 10–20 cm during removal from the CaCl_2_ bath. Thus, alignment depends on optimizing extensional stress and the viscosity of the pre‐gel solution. Viscosity plays a critical role, if too high, it hinders steady flow; if too low, the solution disperses in the CaCl_2_ bath and fails to form stable noodles. Hence, parameters such as needle diameter, flow rate, and solution viscosity must be carefully optimized to achieve good fibrillar alignment.

The macroscopic alignment of the gel noodles was further analyzed using 2D small‐angle neutron scattering (SANS), as small‐angle scattering is an excellent tool to capture the macroscopic fibrillar orientation [12, 19, 24]. Gel noodles of thick and thin diameters were cut and positioned lengthwise on a zig‐zag folded thin aluminum foil for measurement (Figure 2d,e). These were compared with bulk gels to assess fibrillar orientation. The 2D SANS results for 2NapFF bulk gel showed isotropic scattering (Figures S11 and S12), indicating randomly oriented fibrils. In contrast, the gel noodles displayed anisotropic scattering patterns, suggesting that the fibrils were predominantly aligned along the noodle length. Azimuthal intensities across all φ directions were extracted from the 2D SANS patterns [52], showing two peaks at ∼0° and ∼180°. These profiles were fitted using a two‐peak Gaussian model (Figure 2f; Figures S13–S17), and the peak intensities were used to assess fibril orientation. Anisotropy was quantified from the relative peak‐to‐baseline intensities using a previously reported method (Equations S1 and S2) [50]. The results showed that for all gelators, the thin segments had higher degrees of anisotropy than the thick segments (Table 1). This confirms that for all gelators tested, the thinner segment of any noodle exhibits superior macroscopic alignment with fibrils oriented along the length. However, the difference in alignment between thick and thin segments varied across gelators. For 2NapFF and 4BPFF, alignment improved substantially in the thin segments compared to the thick ones. For other gelators, the improvement was less pronounced. 2NapFF and 4BPFF were also the two systems where thin segments formed most efficiently. These observations suggest a qualitative design map: molecular substitutions that promote long, cohesive cylindrical micelles at high pH (and measurable extensional relaxation) favor macroscopic alignment on Ca^2+^ cross‐linking. The structure–rheology–alignment relationships offer practical guidelines for gelator selection and provide the platform on which predictive models can be built.

**TABLE 1:** Calculated anisotropy in the thick and thin segments of different gel noodles, with small values representing low alignment and values close to 1 indicating high alignment.

Segment	2NapFF	*LD*‐2NapFF	1ThNapFF	4BPFF	1PyrFF	2AnqFF
Thick	0.24 ± 0.03	0.77 ± 0.01	0.70 ± 0.01	0.52 ± 0.01	0.31 ± 0.02	0.70 ± 0.01
Thin	0.94 ± 0.00	0.89 ± 0.00	0.84 ± 0.00	0.96 ± 0.01	0.58 ± 0.02	0.83 ± 0.01

**FIGURE 3:**
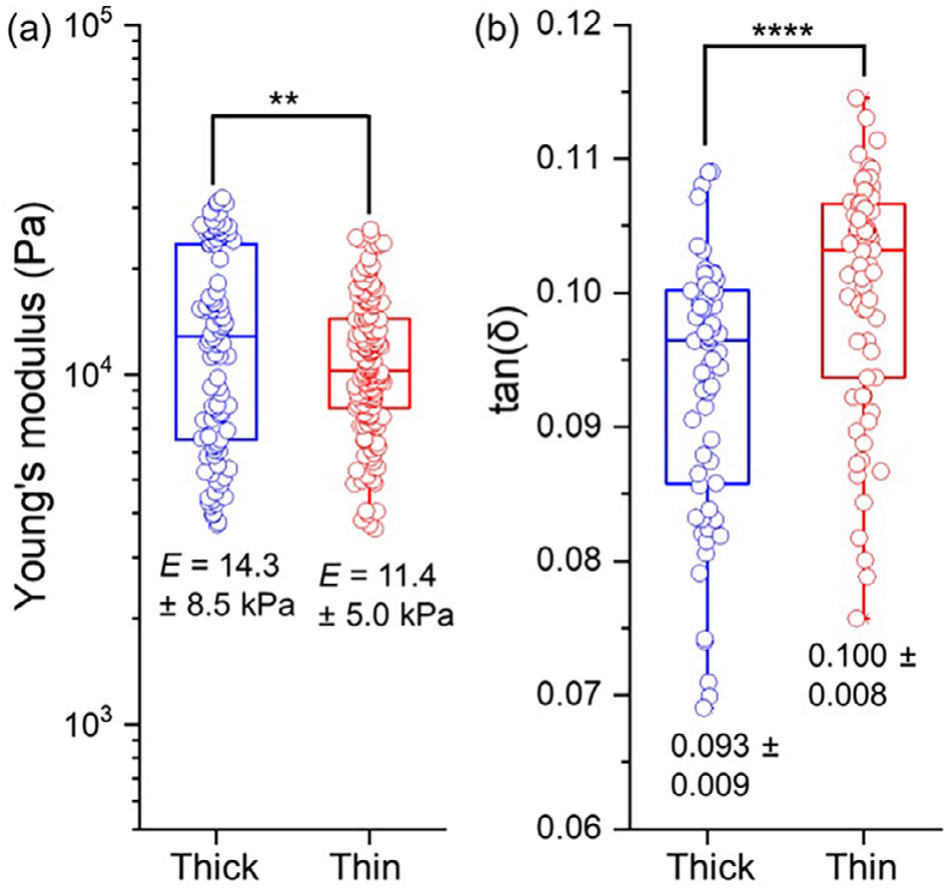
(a) Nanoindentation performed on thick and thin segments of 2NapFF noodles. (b) Viscoelastic analysis on the same (*n* ≈ 50). Statistical significance was assessed using Welch's *t*‐test in MATLAB (^**^0.001 < *p* ≤ 0.01, ^****^ *p* <0.0001).

We compared the elasticity and stiffness of the thick and thin segments of a representative noodle made from 2NapFF. Due to their 1D structure, standard bulk rheological measurements were not possible. Instead, nanoindentation was used to assess local mechanical properties at the micron scale (Figure S18). Three independent noodle samples were prepared, and one thick and one thin segment were cut from each sample. Indentation data were collected from two distinct regions on each segment, yielding a total of six matrix scans. Both the thick and thin segments showed comparable (** 0.001< *p* ≤ 0.01) Young's modulus values (Figure 3a). This was expected, as both segments were formed from the same pre‐gel and calcium chloride solutions. The slightly higher Young's modulus observed in the thick segments can be attributed to lower anisotropy, indicating that fibrils may be oriented in multiple directions. This broader distribution of fibril orientations likely enhances cross‐linking within the network, resulting in increased local stiffness in the thick noodles.

We also assessed the viscoelastic properties of the thick and thin segments of 2NapFF noodles using the same nanoindentation setup. Dynamic mechanical analysis was performed at two regions on each of three noodles, resulting in six matrix scans. The noodles were indented to a consistent depth and subjected to oscillatory loading across a range of frequencies (1, 2, 4, 10 Hz), all within the linear response regime. The tan(𝛿), calculated as the ratio of loss modulus (*G*″) to storage modulus (*G*′), was determined from the phase shift between stress and strain over time. The tan(𝛿) of the 2NapFF thick segments was slightly lower than that of the thin segments (^****^ *p* <0.0001, Figure 3b), indicating greater elastic behavior at the micron scale, which is in line with the slightly higher Young's modulus observed.

We further investigated the bulk mechanical properties of the gel noodles by evaluating their axial stiffness through tensile testing. The thick and thin segments of each noodle were mounted vertically with a grip‐to‐grip distance of 2 cm and stretched at a rate of 5 mm/min until rupture. For 2NapFF noodles, despite having diameters approximately 20 times smaller, the thin segments withstood similar or greater tensile forces before breaking compared to the thick segments. Stress–strain curves were generated by converting the recorded force into nominal stress, calculated by dividing the force by the initial cross‐sectional area. Since stress is highly dependent on the noodle's cross‐sectional area, we accurately measured the diameters of thick and thin segments using the Canny edge detection function in MATLAB (Figures S19 and S20). Pixel dimensions were converted to length using the microscope calibration factor, and the cross‐sectional area was calculated assuming the noodles to be cylindrical. Most thick segments had a consistent diameter of ∼0.6 mm, determined by the fixed needle diameter. In contrast, the thin segment diameters varied between 30–120 µm, which is expected as their dimensions depend on the solution properties and extensional viscosity.

**FIGURE 4:**
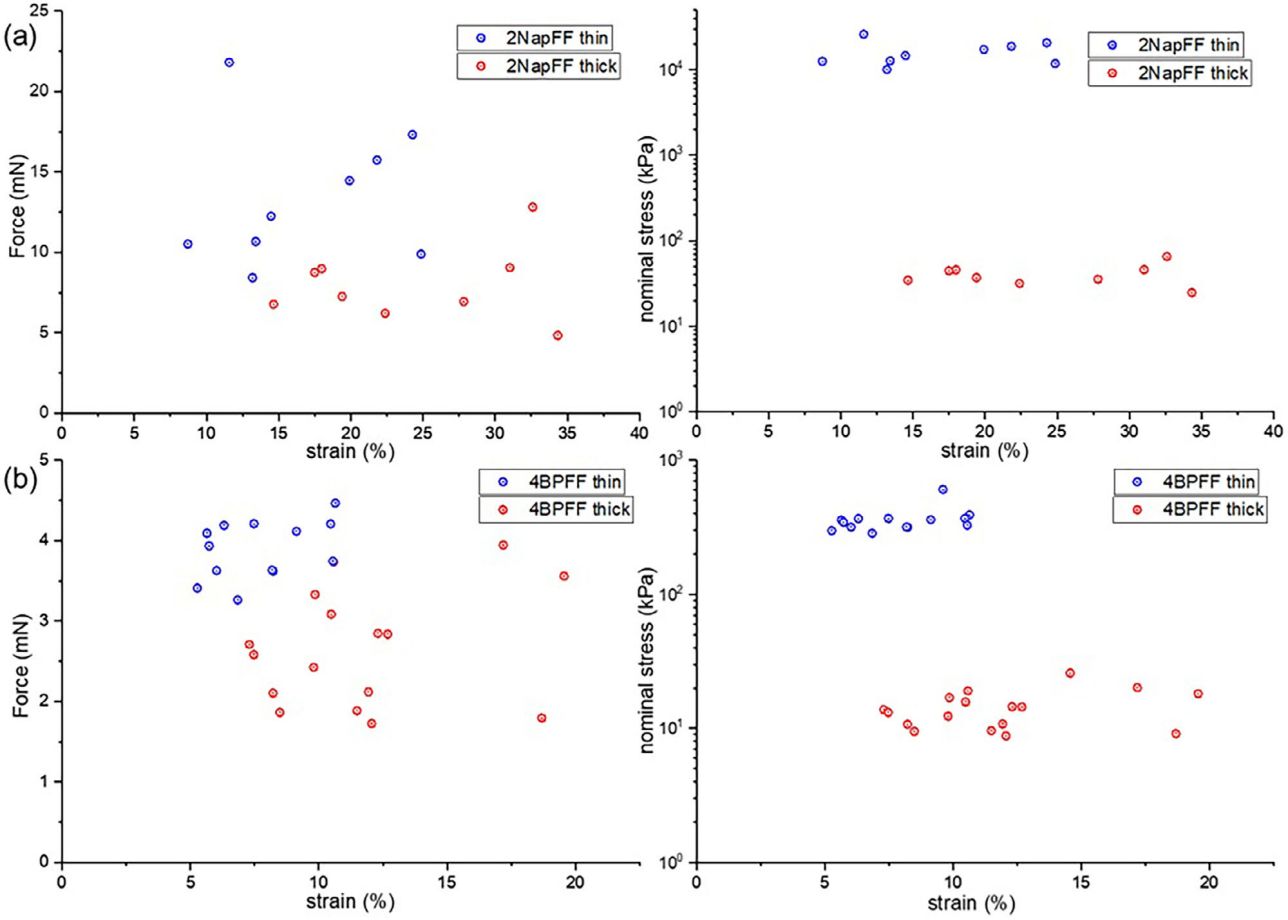
The force vs. strain (left) and nominal stress vs. strain (right) at the breaking point for thick (red) and thin (blue) gel noodles: (a) 2NapFF, (b) 4BPFF. Each data point represents the force (or stress) and corresponding strain at the rupture point of an individual tensile test.

The resulting stress–strain curves confirmed that the 2NapFF thin noodles typically exhibited 400‐fold greater resistance to tensile stress (Figure 4a), reaching up to 10–20 MPa of stress resistance. In contrast, the thick noodles exhibited a higher ultimate tensile strain capacity, which was expected due to their larger diameter. Ultimate tensile strain, which represents the extent to which a material can be stretched before rupture, typically increases with diameter. This is because larger‐diameter materials can generally accommodate more deformation before breaking. However, it was surprising that the thick noodles, despite their 20‐fold larger diameter, only withstood about twice the strain of the thin noodles. The large difference in nominal stress at failure is strongly amplified by the smaller cross‐sectional area of the thin segments, and alignment further contributes by increasing the fraction of load borne along the noodle axis. The thin segments formed under the pump‐off regime exhibited pronounced alignment of the internal fibrils along the noodle axis. Since the tensile force was applied along the same direction as the fibril alignment, the anisotropic architecture likely improves efficient load transfer along the fibrillar network, enhancing tensile strength. In contrast, the thick segments, which displayed less alignment, lacked this structural coherence, resulting in reduced stress resistance. These results demonstrate that processing‐induced alignment increases axial load‐bearing efficiency and that the large increase in nominal stress at failure arises from the combined effects of reduced cross‐sectional area and higher retained orientation.

The tensile data collected from all gel noodles showed that the force resistance of the thick and thin segments was comparable. However, due to differences in diameter, their stress resistance varied significantly, scaling with the square of the diameter difference. Among the thick segments, only 1ThNapFF showed stress resistance similar to that of 2NapFF. In contrast, *LD*‐2NapFF, 4BPFF, and 1PyrFF noodles were much more fragile, with the thick segments withstanding only about 5–10 kPa of stress (Figure 4b; Figures S21–S23). The thin segments of 2AnqFF noodles were too short and fragile to be mounted on the tensile testing setup, either failing to reach the required length for wrapping or breaking during mounting. As a result, we were unable to obtain tensile data for the thin noodles of 2AnqFF. Analysis of the tensile data from the other thin noodles showed that 1ThNapFF noodles could withstand stresses of ∼1 MPa due to their relatively higher force resistance. In contrast, the thin noodles of *LD*‐2NapFF, 4BPFF, and 1PyrFF ruptured at stresses between 200–400 kPa.

It is important to note that the mechanical properties assessed by nanoindentation and tensile tests represent different properties of the material. First, nanoindentation measures local stiffness at the micron scale, primarily governed by micellar‐level cross‐linking between 2NapFF tubes and Ca^2+^. In contrast, tensile testing evaluates bulk mechanical properties, where self‐assembly across multiple length scales contributes to the response. Since the molecular‐level aggregation is the same in both noodle segments, the higher tensile stability of the thin noodles can be attributed to their improved fibrillar alignment. Accordingly, we interpret the higher axial tensile strength as a load‐sharing effect of oriented fibrils, where better alignment puts more fibrils along the loading axis and improves load transfer without necessarily changing the local indentation modulus. Furthermore, tensile testing applies force along the length of the noodle, whereas nanoindentation applies force perpendicular to it (Figure S18). As a result, higher anisotropy along the noodle axis enhances tensile strength but may not increase the Young's modulus measured by indentation.

Aligned supramolecular gels are promising candidates for guiding cellular behavior and directional tissue engineering, such as muscle cell regeneration [21, 22, 25, 58, 59]. While they have shown important cell‐guidance capabilities, many reported systems do not demonstrate uniform macroscopic alignment over extended lengths, which can constrain translation to scaffold formats. For example, previous studies have shown that while peptide‐based gels can support cell growth, isotropic architectures fail to provide directional cues necessary for organized cell orientation and function [60]. Our system overcomes this limitation by producing thin, highly aligned gel noodles in the tail segments with well‐defined anisotropy and tensile integrity. These features make the noodles as a potential contact‐guidance substrate for anisotropic cell alignment, such as muscle [23], while recognizing that functional tissue engineering performance requires broader biological validation. To explore this potential, we investigated the response of C2C12 myoblasts on aligned vs. non‐aligned gel noodles. We studied cell culture on both thick and thin segments to evaluate the impact of enhanced alignment on cellular behavior. Initial experiments were conducted with 2NapFF noodles, which showed excellent tensile stability and distinct alignment differences between thick and thin segments. However, the study was not possible as both segments fully dispersed in cell culture media within two days. As a result, we proceeded with 4BPFF noodles, which also exhibited clear alignment differences between the segments (Table 1; Figure S15) and remained stable under culture conditions.

**FIGURE 5:**
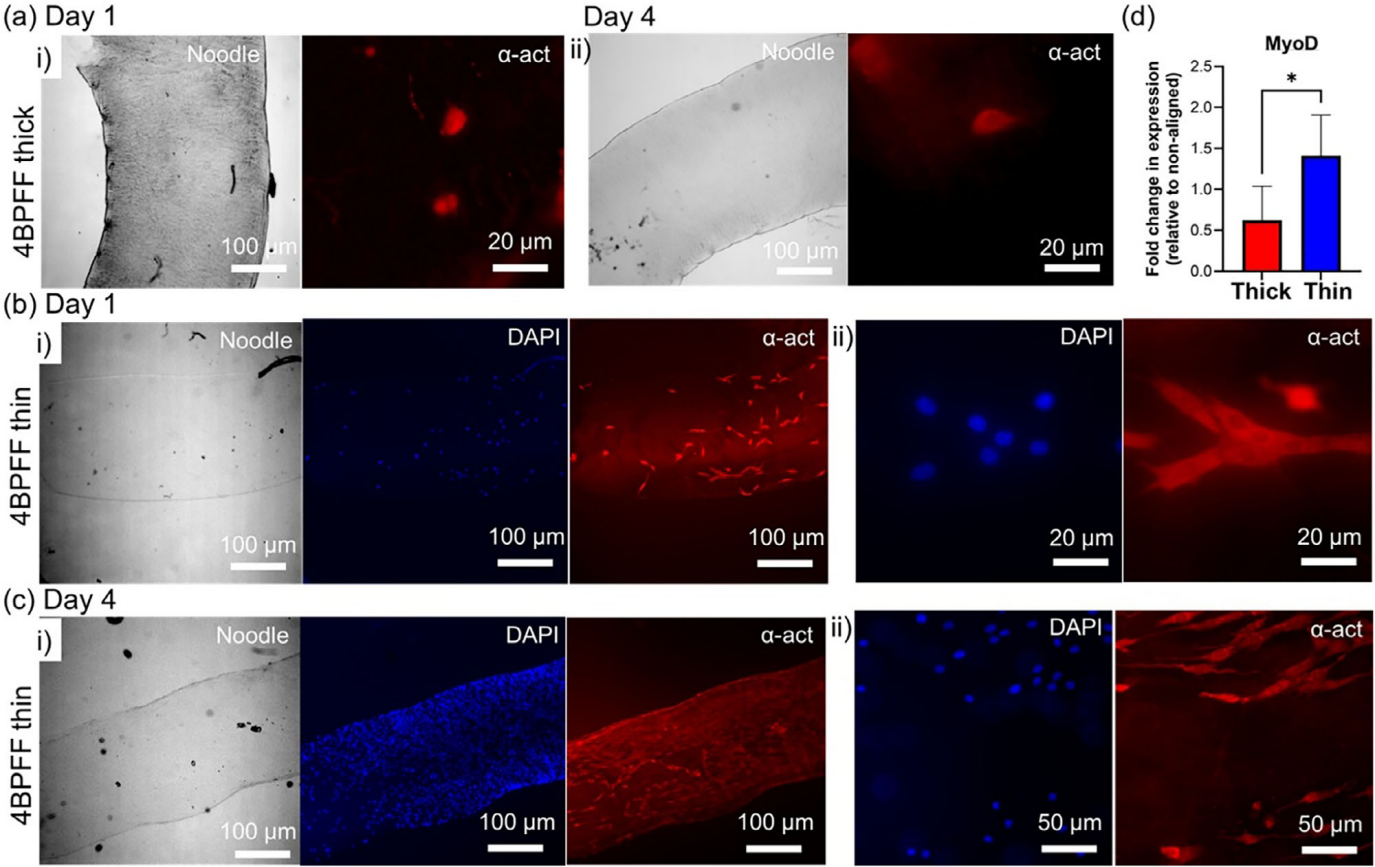
Aligned 4BPFF noodles promote C2C12 elongation, interconnectivity, and myogenesis: (a) C2C12 cells on thick segments of 4BPFF noodles after (i) 1 and (ii) 4 days of culture. (b) C2C12 cells on thin segments of 4BPFF noodles after 1 day at (i) low and (ii) high magnification. (c) C2C12 cells on thin 4BPFF noodles after 4 days at (i) low and (ii) high magnification. (d) qPCR for the early myogenic transcription factor MyoD after culturing for 4 days on thick and thin 4BPFF noodles; data were normalized to housekeeping gene GAPDH and further normalized to the thick noodle sample (*n* = 3, * = *p* ≤ 0.05). In all images, 4BPFF noodles were visualized using brightfield, and cells were stained with DAPI (nuclei) and α‐actinin (sarcomeric structural marker). The noodles had been placed between two slides during imaging meaning true thickness could not be captured, and image quality was limited by sample opacity and reduced light transmission.

We characterized C2C12 adhesion and morphology, including elongation and orientation, on 4BPFF noodles with contrasting macroscopic alignment, and profiled early myogenic gene expression as a molecular readout. In Figure 5a, we could see on non‐aligned noodles that cells displayed a very rounded morphology, suggesting weak adhesion, and isolated cells with no evidence of proliferation or cell–cell interconnectivity when stained for the myogenic marker α‐actinin after 1 (**i**) and 4 (**ii**) days of culture; DAPI staining for nuclei was challenging to acquire on thick segments.

By contrast, on the thin noodles, cells were significantly more elongated along the material within 24 h (Figure 5b) as quantified by an orientation analysis (Figure S24), and after 4 days formed interconnected cells that aligned along the axis of the noodle (Figure 5c). Starting from a low seeding density, the cells expanded over several days to cover the entire surface, as seen in the microscope images. We performed qPCR and observed upregulation of myogenic marker MyoD for cells on aligned (thin) noodles after 4 days of culture (Figure 5d); this coincides with a more elongated and interconnected phenotype visualized in Figure 5c. Consistent with this role, the increased MyoD on the thinner, more aligned noodles suggests that substrate alignment may trigger early, material‐dependent myogenic signaling under growth conditions, as reported in previous studies [59, 61]. Collectively, these preliminary data indicate that C2C12 cells attach and align more readily on the thinner, more aligned noodles and exhibit increased early myogenic gene expression, supporting the concept that macroscopic alignment of the scaffold can act as an instructive cue. Future studies will incorporate quantitative cytoskeletal analyses to complement morphology and gene expression.

These results suggest that alignment within supramolecular gel noodles can influence both mechanical performance and cellular contact guidance under the conditions tested. The ability to fabricate anisotropic gel noodles through a simple processing‐history approach enables a platform for studying how supramolecular alignment influences mechanics and cell contact guidance. In particular, the aligned noodles support preliminary myoblast attachment and elongation and are presented here as proof of compatibility and directional influence, providing a basis for future studies toward muscle regeneration and tissue engineering. The reproducibility of this alignment across multiple gelators, along with demonstrated stability under culture conditions, suggests that this strategy can be extended to design next‐generation scaffolds with tunable architecture. Previous systems including aligned peptide amphiphile tubes [27], modular hydrogel channels [47], and electrospun fibrous scaffolds [33], have successfully guided cell alignment, but often rely on complex fabrication, static geometries, or external templates [62]. In contrast, our shear‐based method yields freestanding, aligned gel noodles using simple extrusion, offering modularity, mechanical anisotropy, and aqueous stability. The most aligned segments arise during a brief, pressure‐relaxation burst rather than continuous flow, limiting maximum aligned length. In principle, step‐and‐release protocols or controlled back‐pressure could extend aligned segments, but such process engineering lies outside our current scope. While current gel noodles are yet to incorporate bioactive features or support cell encapsulation, they provide a versatile platform that could be enhanced with biochemical functionalization and structural tuning for broader tissue engineering applications.

## 3 Conclusion

We have demonstrated a processing‐history‐based fabrication approach for generating 1D supramolecular gel noodles with enhanced fibrillar orientation. By implementing a two‐stage extrusion protocol (pump‐on extrusion followed by pump‐off pressure‐relaxation driven extrusion), we produced extended thin segments of noodles with distinct flow histories. These segments exhibited significantly higher anisotropy compared to their thicker counterparts, as confirmed by POM and SANS. Mechanical analysis showed that the thin segments exhibited markedly higher nominal stress at failure, reflecting the combined effects of reduced cross‐sectional area and higher retained alignment, underscoring the role of macroscopic orientation in axial load‐bearing. The strategy was effective across a range of dipeptide‐based gelators, with 2NapFF and 4BPFF showing the most significant improvement in fibrillar anisotropy. The aligned noodles supported preliminary C2C12 myoblast attachment and elongation and showed increased MyoD expression. These results highlight the potential of aligned supramolecular gel noodles as contact‐guidance substrates and advance future studies toward functional anisotropic tissue models. The simplicity and scalability of this method make it a valuable tool for designing anisotropic soft materials and probing how processing‐controlled alignment affects mechanics and cell response.

## 4 Experimental

### 4.1 Materials and Methods

All chemicals were purchased from Sigma–Aldrich or Fluorochem Ltd and used as received. All the gelators used are reported FF‐based derivatives and were synthesized as previously described [20, 28, 54, 63, 64]. The crosslinker CaCl_2_ was purchased from Fluorochem, and a 0.5 M solution was freshly prepared before noodle formation. Deionized water was used in all experiments.

### 4.2 Preparation of the Dipeptide Solutions

200 mg of the dipeptides was taken in a 14 mL glass vial, and 1 equivalent of NaOH (0.1 M solution), and the required amount of water was added to attain a final volume of 5 mL. The mixtures were stirred overnight, and the pH of the solutions was adjusted to 10.5 ± 0.05 with 1.0 M NaOH solution. For thermal annealing of 1PyrFF and 2AnqFF solutions, the vial was wrapped with Al‐foil to prevent any water evaporation, then placed in an oven at 60°C for 1 h. After 1 h, the vial was kept at room temperature for cooling. If needed, the pH was readjusted with 1.0 M NaOH solution.

### 4.3 Viscosity Measurements

The viscosity measurements were performed in an Anton Paar Physica MCR101 rheometer using a 50 mm cone geometry (CP50) with a cone angle of 1°. The instrument‐defined truncation gap between the geometry and the plate was 0.101 mm and the temperature was kept at 25°C. ∼1 mL of each sample was poured onto the plate to avoid any shearing caused by pipetting the solutions. All viscosity measurements were carried out in triplicate, and the mean values and standard deviations were reported.

### 4.4 Extensional Viscosity Measurements

Extensional relaxation times (*λ_E_*) were determined using the dripping‐onto‐substrate (DoS) method, following a previously reported protocol [56, 57]. Briefly, each sample was loaded into a 10 mL syringe connected to a 19G flat‐headed needle and mounted on a ProSense syringe pump. Fluids were dispensed onto a 4 mm diameter cylindrical glass substrate at a controlled flow rate of 3 mL/min. Dispensing was stopped immediately before droplet contact to initiate formation of a liquid bridge. The thinning and break‐up dynamics of the resulting fluid filament were recorded at 60 frames per second using a standard mobile camera.

Recorded video files of the extensional viscosity experiments in .mp4 format were processed using a custom Python script employing OpenCV (v4.5.5). Each video was automatically converted into a series of uncompressed TIFF images, with one image extracted per frame. The script systematically created a separate output folder for each video, named according to the original filename, and saved the corresponding frames within.

The TIFF image series was analyzed using MATLAB R2024b. A custom script employed the Canny edge detection algorithm to extract the filament profile from each frame and calculate the filament diameter over time. Frames were sorted chronologically, and the diameter of the liquid bridge was computed based on the distance between edge pixels near the top and bottom of the filament. A moving average filter (window size = 15 frames) was applied to the diameter data to reduce noise. Outliers were removed using a 3σ criterion centered around the median diameter.

To extract the relaxation time (*λ_E_*), the filament thinning curve was manually segmented to isolate the elastocapillary regime, where exponential thinning dominates. The selected region was fitted in OriginPro 2018 using a custom user‐defined fitting function based on the theoretical model:

(1) $$D\left(t\right)=D_{0}\cdot \exp\left(\frac{-t}{3\lambda_{E}}\right)+D_{\infty }$$

where D_0_ is the initial diameter offset, *λ_E_* is the relaxation time, and D_∞_ accounts for the residual diameter (baseline) at long times. The fitting returned *λ_E_* along with associated confidence intervals and goodness‐of‐fit metrics (*R^2^*, reduced chi‐squared), ensuring the reliability of the extracted values.

### 4.5 Preparation of Gel Noodles

Gel noodles were prepared by extruding the pre‐gel solution (40 mg/mL in water, pH 10.5 ± 0.05) from a 10 mL luer‐lock syringe (plastic barrel, flat‐end 21G stainless steel needle) mounted on a ProSense syringe pump at 3 mL/min into ∼20 mL of freshly prepared 0.5 M CaCl_2_ contained in a 90 mm petri dish rotating at 100 rpm (clockwise) on an Ossila spin coater. After 15–20 s of pumping to initiate the ‘thick’ segment of the noodle, the pump was stopped to allow transient internal back‐pressure in the syringe barrel to relax; this residual pressure, together with the solution's extensional viscosity, drove a slower, self‐thinning extrusion that produced the ‘thin’ tail segment (Figure 2a,b; see also Figure S3). Using fixed hardware (syringe volume and needle gauge) and a defined pre‐gel concentration/pH made the pressure‐relaxation step reproducible across runs; thin‐segment lengths were typically 15–30 cm, and diameters in the 30–120 µm range depending on the peptide composition (Figures S4–S9).

To improve reproducibility, we standardized syringe geometry (10 mL luer‐lock), needle length (18 mm) and diameter (21 G), pump flow (3 mL/min), rotation speed of the spin coater (100 rpm), and pump stop time (15–20 s) before the pressure‐relaxation step. Under these fixed conditions, thin‐segment formation was observed in all the gelators but with variable length and diameter, as the absolute barrel pressure depends on solution viscosity and plunger friction.

### 4.6 Polarized Optical Microscopy

Microscopic images were captured using a Leica DM750 microscope at 5x–10x magnification. Initially, the white light was calibrated, and brightfield images were captured. Then the polarized light was turned on and the orientation of the polarizer was adjusted to 90° to capture polarized light images. The scale bars were inserted using ImageJ software v.1.54h (National Institutes of Health, Maryland, USA) [65].

### 4.7 Small Angle Neutron Scattering (SANS)

For the SANS experiments, the dipeptide pre‐gel solutions and 0.5 M CaCl_2_ solution were prepared in a NaOD/D_2_O medium, then the noodle was prepared as described above. To load the noodles in Hellma cuvettes, a thin sheet of Al‐foil was cut and folded into a zig‐zag pattern. About 2 cm length from either the thick or the thin segment of the noodle was then cut using fine scissors to minimize axial disturbance and positioned along the length of the folded Al‐foil, with a total of 6–10 filaments placed within the foil. The prepared foil was carefully inserted into a Hellma cuvette (2 mm path length), which was then filled with D_2_O and sealed. SANS experiments were carried out on the SANS2d instrument at the ISIS Neutron and Muon source (STFC Rutherford Appleton Laboratory, Didcot, UK) with a source‐to‐sample and sample‐to‐detector distance = 12 m, and neutrons of wavelength range 1.75–12.5 Å to access a Q range from 0.00154 to 0.49 Å^‒1^. The resulting data were converted to a 2D scattering diagram using the Mantid data reduction software [66]. This process included, but was not limited to, corrections for detector efficiencies, normalizing the full detector images, and subtracting background scattering from a cell containing D_2_O. The instrument‐agnostic data were then analyzed using the SasView software v.5.0.6 [67].

### 4.8 Nanoindentation

Nanoindentation measurements were performed using a Chiaro nanoindentation device (Optics11, NL), mounted on top of an inverted Zeiss Axiovert 200 M microscope, following a previously described protocol [68]. A probe of stiffness (*k*) 0.48 N m^−1^ was used for the measurements. The radius (*R*) of the spherical tip attached to the cantilever was 3 µm. Approximately 3 cm of the gel noodle was cut with scissors and placed in a glass petri dish. A metal washer was placed on the top of the sample to prevent movement. Deionized water was added to fully immerse the sample and prevent drying. The petri dish was placed on the microscope stage, keeping the sample along the X‐direction. At least two matrix scans were performed on each sample, and each matrix scan consisted of 25 indentations. The spacing between subsequent indentations was 6 µm. For data analysis, the forward segment of the collected force‐displacement (*F*‐*z*) curves were analyzed using a custom open‐source software [68]. The contact point was identified by a goodness of fit algorithm to convert *F*‐*z* curves into force‐indentation (*F*‐*δ*) curves. The *F*‐*δ* curves were analyzed up to a maximum indentation of *δ* = 0.1*R*, which is indeed much smaller than the thickness of the thin gel noodle (typically 30 µm). This regime justifies fitting the data with the Hertz model for the indentation of a rigid sphere over a half plane and ignoring any contribution of the bottom rigid substrate [68].

### 4.9 Viscoelastic Measurements

Viscoelastic measurements were performed via dynamic mechanical analysis (DMA) using the same device as nanoindentation measurements. A probe of stiffness (*k*) 0.52 N m^−1^ was used for the measurements. The radius (*R*) of the spherical tip attached to the cantilever was 3.5 µm. Similar to nanoindentation measurements, the noodle segment was immobilized with a metal washer in a petri dish and submerged in deionized water to prevent drying. The petri dish was placed on the microscope stage and matrix scans were performed along the *x*‐axis of the material. An indentation depth of 3 µm was achieved using a loading speed of 5 µm/s before an initial relaxation time of 30 s. DMA frequencies of 1, 2, 4, and 10 Hz were used with 5 periods per frequency at an amplitude of 300 nm, and 2 s relaxation pauses in‐between frequencies. Tan(*δ*) was derived using Optics11Life DataViewer v2.7 analysis software.

### 4.10 Determining the Noodle Diameter

The noodles were imaged on the Leica DM750 microscope at 5x magnification as described before. Diameters of the noodles were measured from these optical images using a custom MATLAB (v2024b) script. Each image was first converted to grayscale and smoothed using a Gaussian filter (*σ* = 2) to reduce background noise. A vertical intensity gradient was then computed to identify sharp changes in contrast along each image column. For each column, the position of the strongest negative gradient (dark‐to‐light transition) was taken as the top edge, and the strongest positive gradient (light‐to‐dark transition) as the bottom edge, thereby defining the boundaries of the noodle. To minimize noise and local fluctuations, both edge profiles were further smoothed using a combination of median filtering, moving average, and LOWESS (locally weighted scatterplot smoothing). A total of 50 positions were then sampled uniformly along the width of the noodle. At each position, the vertical distance between the top and bottom edges was calculated to obtain the local diameter. The resulting 50 diameter values (in pixels) were visually inspected, and any outliers resulting from image artifacts (e.g. dust or background contrast) were excluded manually. The final mean diameter and standard deviation were calculated from the remaining valid measurements. The diameters (in pixel) were then multiplied by the microscope calibration factor to obtain the diameter in µm.

### 4.11 Tensile Testing Experiments

The tensile properties of the noodles were investigated using a Zwick Z2.0 UTM testing machine (Zwick GmbH & Co. KG, Germany), running on TestExpert V1.8 software, and equipped with a 5 N load cell (on top). All measurements were performed with wet noodles to preserve the hydrated state. A ∼5 cm long noodle was cut from either the thick or thin segment, and was mounted onto custom 3D‐printed holders with a grip‐to‐grip separation of 2 cm. The noodle was then stretched uniaxially from the top at a rate of 5 mm/min. The applied force and strain were recorded until the noodle ruptured. The tensile force was then converted to nominal tensile stress (σ_nom_ = F/A_0_) by dividing by the initial cross‐sectional area A_0_, calculated from the noodle diameter (assuming a circular cross‐section) as described before.

### 4.12 Cell Culture

All cell culture reagents were provided by Gibco. C2C12 cells (ATCC, CRL‐1772; passage 20) were cultured using Dulbecco's Modified Eagle Medium (DMEM) supplemented with 10% fetal bovine serum (FBS), 1X non‐essential amino acids (NEAA), 1X penicillin/streptomycin (P/S), 1 mM sodium pyruvate, and 2 mM L‐glutamine. Cells were cultured at 37°C with 5% CO_2_ and ∼95% relative humidity. The cell‐culture study was designed to examine alignment‐dependent morphology and early myogenic signaling (MyoD) under growth conditions, rather than running a full differentiation protocol or comparing proliferation rates against a positive‐control scaffold.

### 4.13 Seeding Cells on Noodles

4BPFF noodles were washed 3× in sterile‐filtered milli‐Q water to remove excess CaCl_2_. C2C12 cells were trypsinized from flasks and counted before seeding onto 4BPFF noodles in multiwell plates at a density of 50 000 cells/cm^2^.

### 4.14 Immunostaining

The samples were washed with milli‐Q water before fixing with 4% formaldehyde for 30 min at 4°C. Then, a milli‐Q wash was done followed by permeabilization in 0.1% Triton X‐100 for 5 min at room temperature. The samples were then washed with milli‐Q water and blocked for 1 h in 1% bovine serum albumin (BSA). After blocking, all primary Ab solutions were prepared in 1% BSA at appropriate dilutions and added to cover the samples before incubating overnight at 4°C. After primary Ab incubations, the samples were washed 3× for 5 min each with 0.5% Tween‐20 prior to the addition of secondary Ab solutions (diluted appropriately in 1% BSA) and then incubation at room temperature in the dark for 1 h. Samples were then washed 3× for 5 min each with 0.5% Tween‐20 before mounting onto glass slides using VECTASHIELD antifade mounting medium with DAPI (Vector Laboratories). Visualization was performed using a fluorescence microscope (Zeiss AxioObserver Z1). Primary antibodies against sarcomeric α‐actinin (Proteintech; lot 00025160; clone 11313‐2‐AP; cat. 11313‐2‐AP; 1:200) and anti‐rabbit Cy3 (Jackson ImmunoResearch; lot 159083; clone Cy3 AffiniPure Goat Anti‐Rabbit IgG (H+L); cat. 111‐165‐003; 1:200) were used. 4BPFF noodle samples dissolve in PBS so all solutions were made up using milli‐Q water.

### 4.15 qPCR

All reagents were provided by QIAGEN unless otherwise stated. Cell lysis and RNA extraction were performed using a RNeasy mini kit. Cells were directly lysed on the surface of the noodles before RNA extraction. RNA quantity and purity were measured using a NanoDrop 1000 (Thermo Scientific) before performing cDNA synthesis using a QuantiTect Reverse Transcription kit. Real‐time qPCR was performed using a model 7500 real‐time PCR machine (Applied Biosystems) using SYBR green reagents from a QuantiFast SYBR Green PCR kit. 4 ng cDNA was used per gene, primers were used at 1 µM concentrations, and GAPDH was used throughout as a housekeeping gene for normalization of fold‐changes in gene expression. The following primers were used: MyoD (Gene ID: 17927; Fw: 5′‐GCACTACAGTGGCGACTCAGAT‐3′, Rev: 5′‐TAGTAGGCGGTGTCGTAGCCAT‐3′), GAPDH (Gene ID: 14433; Fw: 5′‐CATCACTGCCACCCAGAAGACTG‐3′, Rev: 5′‐ATGCCAGTGAGCTTCCCGTTCAG‐3′). The Ct value was used for quantification by the comparative Ct method. Sample values were normalized to the threshold value of housekeeping gene GAPDH: ∆Ct = Ct (gene of interest)—Ct (GAPDH). The Ct value of the control (non‐aligned) was used as a reference. ∆∆Ct = ∆Ct (experiment)—∆Ct (control). mRNA expression was calculated by the following equation: fold change = 2^−∆∆CT^.

### 4.16 Statistical Analysis

For nanoindentation and viscoelastic data, a two‐sided Welch's *t*‐test was performed using MATLAB (v2024b) to assess statistical significance between two groups with unequal variances. The test was applied to compare the mechanical and viscoelastic properties between thick and thin segments of 2NapFF noodles. Data were assumed to be independent and approximately normally distributed. Significance levels were denoted as follows: ns (*p* >0.05), ^*^ (*p* ≤ 0.05), ^**^ (*p* ≤ 0.01), ^***^ (*p* ≤ 0.001), and ^****^ (*p* ≤ 0.0001).

qPCR data are represented as mean ± SD and were analyzed using GraphPad Prism software (version 10.5.0) where normality tests were performed to determine whether to select parametric or non‐parametric tests. Differences were considered significant for *p* ≤ 0.05 (^*^). For *t*‐tests: if normality tests were passed, unpaired *t*‐tests with Welch's correction were performed; if normality tests failed, Mann–Whitney tests were performed.

### 4.17 Quantification of Cell Orientation

Cell orientation was quantified using ImageJ (v1.54p) [65] from fluorescence images acquired on pump‐off (thin) noodle segments. To account for the curvature of the noodle, each image was straightened by tracing the noodle center line with a segmented line selection and applying the ‘straighten’ function of a width spanning the full noodle. The straightened images were thresholded to generate binary masks and cleaned with standard binary operations to obtain individual cell objects. Ellipse fitting was then performed for each object using ‘Analyze Particles’, yielding the major axis, minor axis, and orientation angle of the major axis. Objects with a major/minor axis ratio < 1.5 were excluded (∼10%) because near‐circular shapes do not provide a meaningful orientation axis. For the remaining cells, angles were reduced from the range of 0–180 ° to 0–90 ° using θ = if(θ > 90, 180 − θ, θ), where θ is the ellipse‐fit angle relative to the straightened noodle axis. The resulting θ values were used to generate a 10 ° binned orientation histogram (θ_rel_) to quantify preferential alignment along the noodle axis.

## Funding

Leverhulme Trust (Grant code: RPG‐2022‐324) and EPSRC (Grant code: EP/2534109/1).

## Conflicts of Interest

The authors declare no conflicts of interest.

## Supporting information




**Supporting File**: smll73064‐sup‐0001‐SuppMat.docx.

## Data Availability

The data that support the findings of this study are available from the corresponding author, Dave J. Adams (E‐mail: dave.adams@glasgow.ac.uk), upon reasonable request.
