# Supplementary material for: Shear‐Induced Anisotropic Supramolecular Gel Noodles for Improved Cell Guidance in Polarized Tissue Engineering
Source: Small. 2026 Mar 12;22(26):e13952. doi: 10.1002/smll.202513952 (PMC13155044; doi:10.1002/smll.202513952)
Supplement: Supplementary file 1 — Supporting File: smll73064‐sup‐0001‐SuppMat.docx. [file SMLL-22-e13952-s001.docx]

Supporting Information

Shear-Induced Anisotropic Supramolecular Gel Noodles for Improved Cell Guidance in Polarized Tissue Engineering

Dipankar Ghosh, Matthew Walker, Lauren Matthews, Charlie Patterson, Oana Dobre, Massimo Vassalli* and Dave J. Adams*


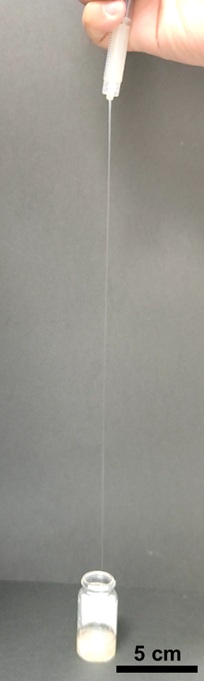


Figure S1: Creating thin strings with 40 mg/mL, pH 10.5 2NapFF solution.


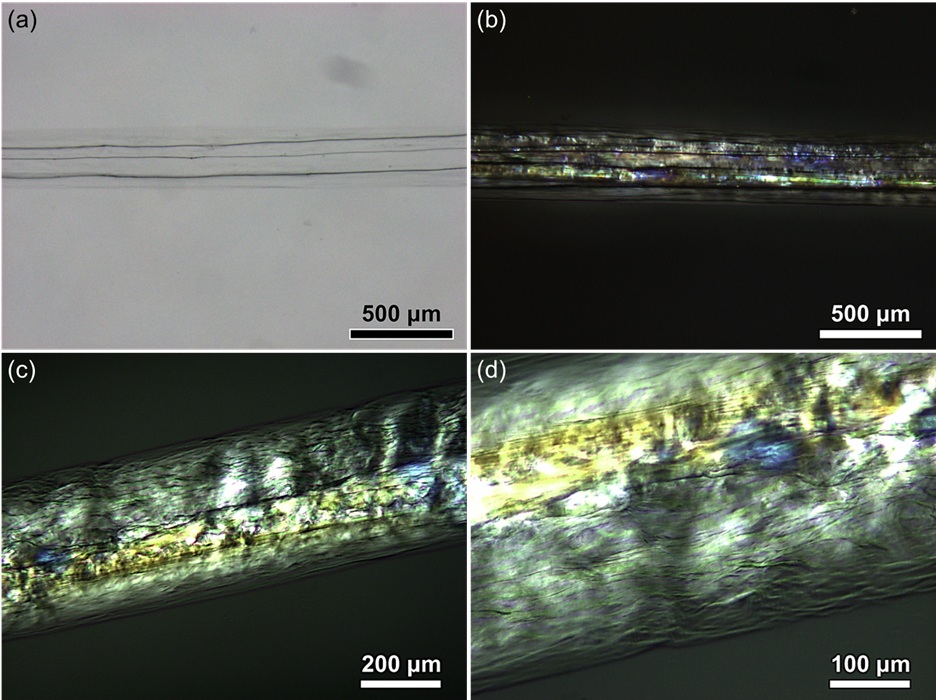


Figure S2: Microscopic images of 2NapFF noodles formed from the string: (a) under brightfield light, and (b-d) and under polarized light at different magnification showing aligned fibrils.


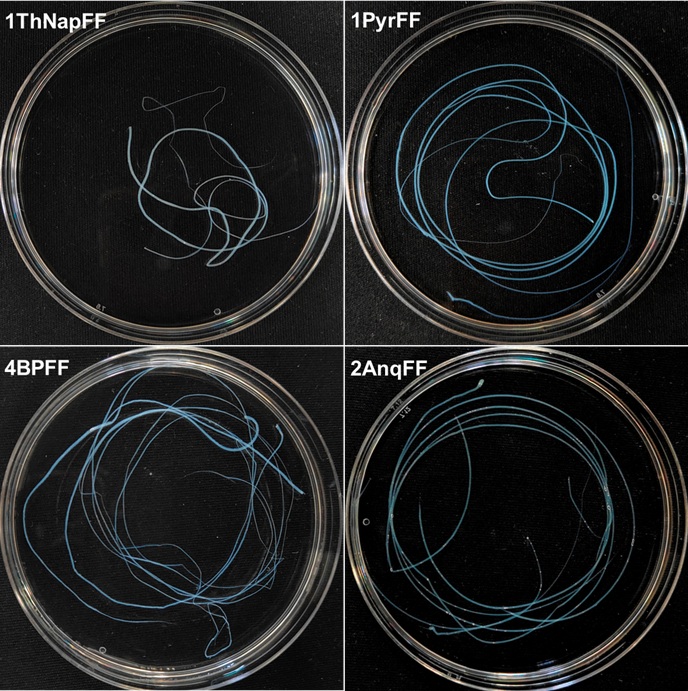


Figure S3: Thick and thin segments of gel noodles formed from different gelators. The diameter of all the petri dishes is 90 mm.


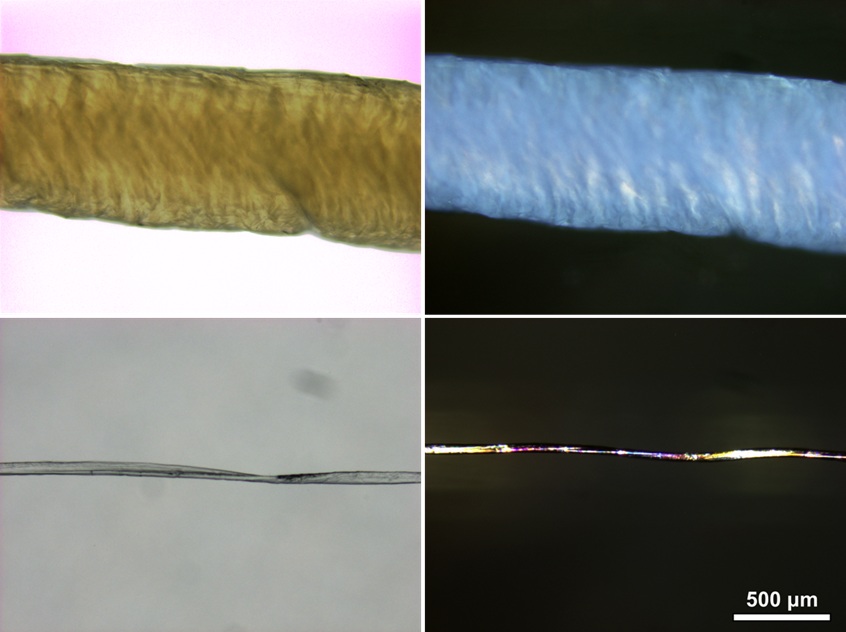


Figure S4: Brightfield (left) and POM (right) images of 2NapFF thick and thin segments. The scale bar is the same for all images.


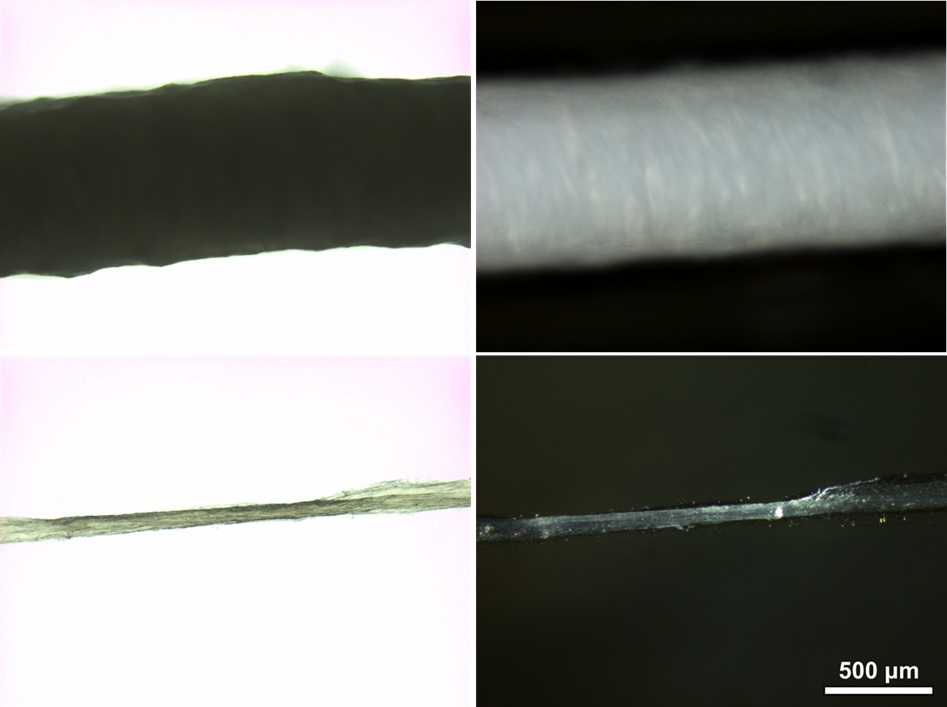


Figure S5: Brightfield (left) and POM (right) images of LD-2NapFF thick and thin segments. The scale bar is the same for all images.


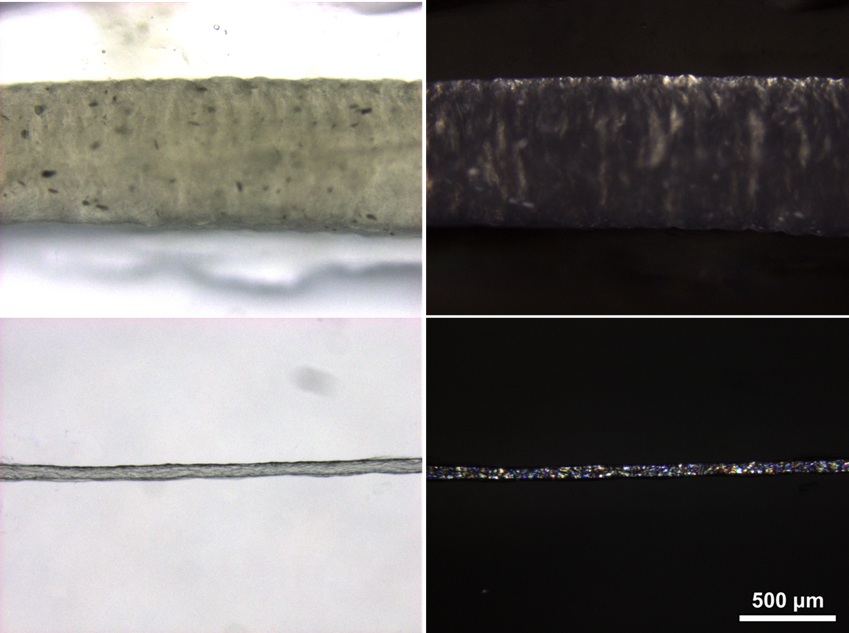


Figure S6: Brightfield (left) and POM (right) images of 1ThNapFF thick and thin segments. The scale bar is the same for all images.


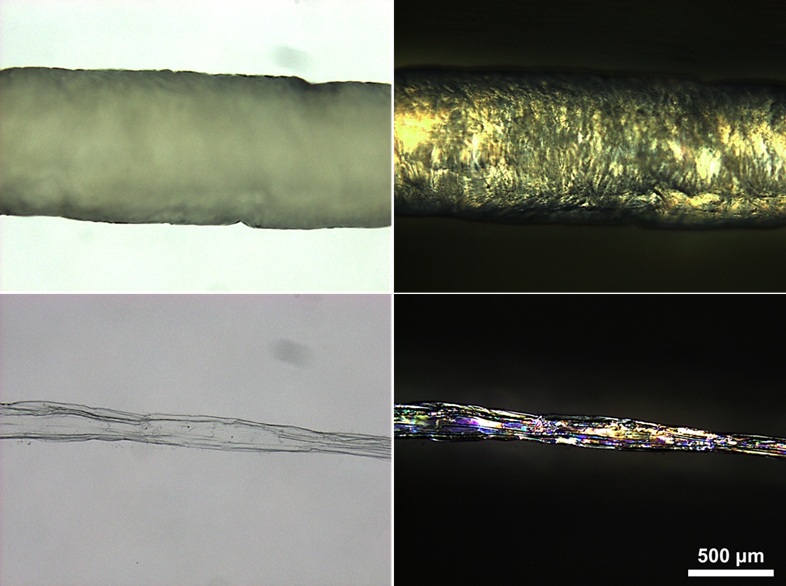


Figure S7: Brightfield (left) and POM (right) images of 4BPFF thick and thin segments. The scale bar is the same for all images.


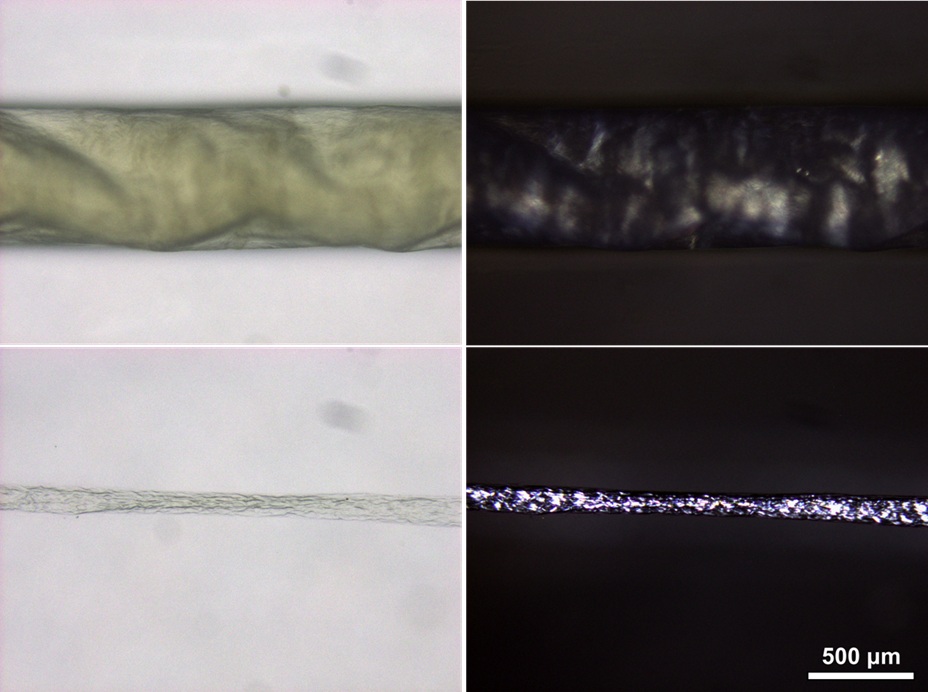


Figure S8: Brightfield (left) and POM (right) images of 1PyrFF thick and thin segments. The scale bar is the same for all images.


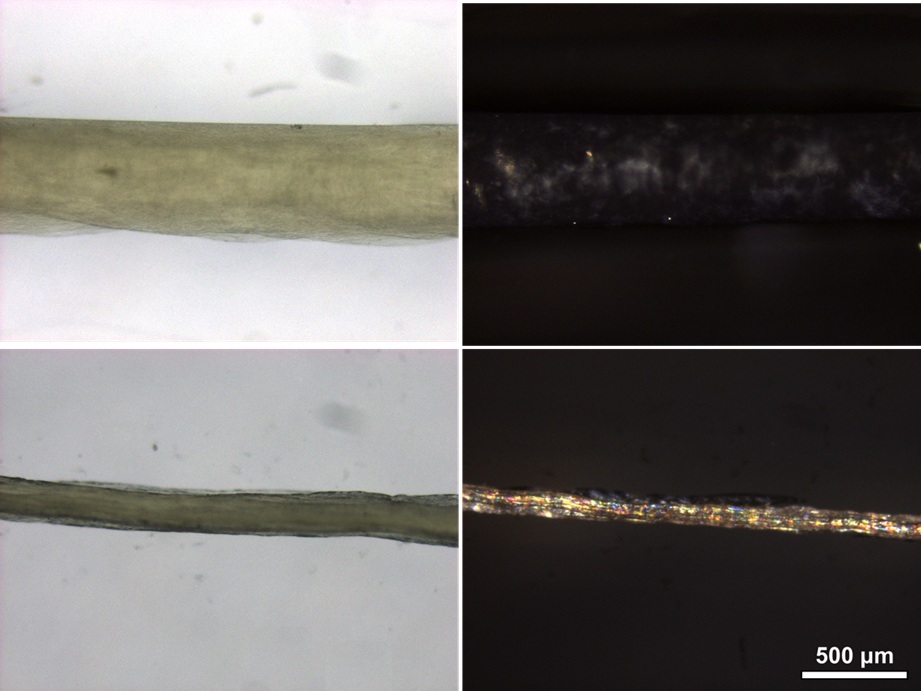


Figure S9: Brightfield (left) and POM (right) images of 2AnqFF thick and thin segments. The scale bar is the same for all images.


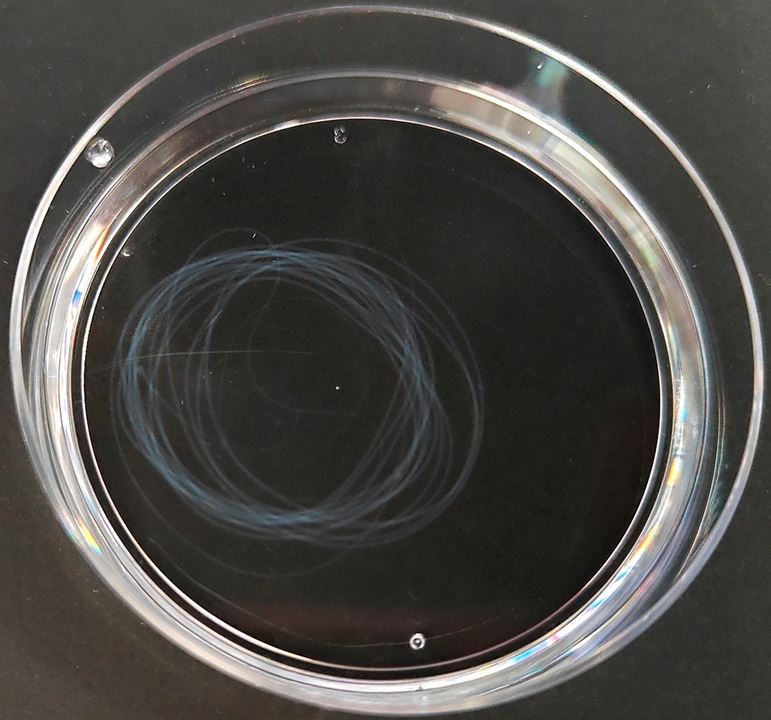


Figure S10: Fragile noodle formed by extruding 2NapFF into CaCl_2_ bath with a 25 G needle. The diameter of the petri dish is 90 mm.


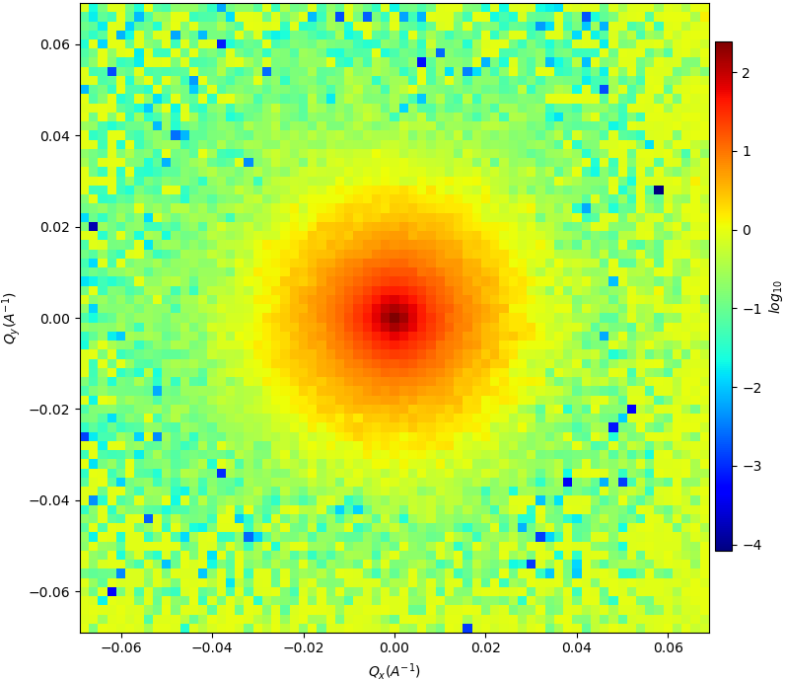


Figure S11: 2D SANS data for 2NapFF + CaCl_2_ bulk gel showing isotropic scattering.


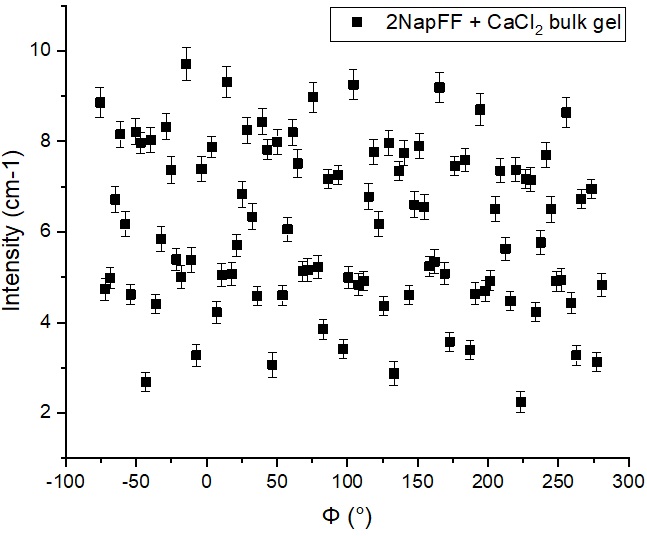


Figure S12: The azimuthal intensity extracted from the 2D SANS data of the bulk 2NapFF + CaCl_2_ gel. The intensity is uniformly distributed across all azimuthal angles (ϕ), indicating an isotropic scattering pattern and making it unsuitable for Gaussian fitting.


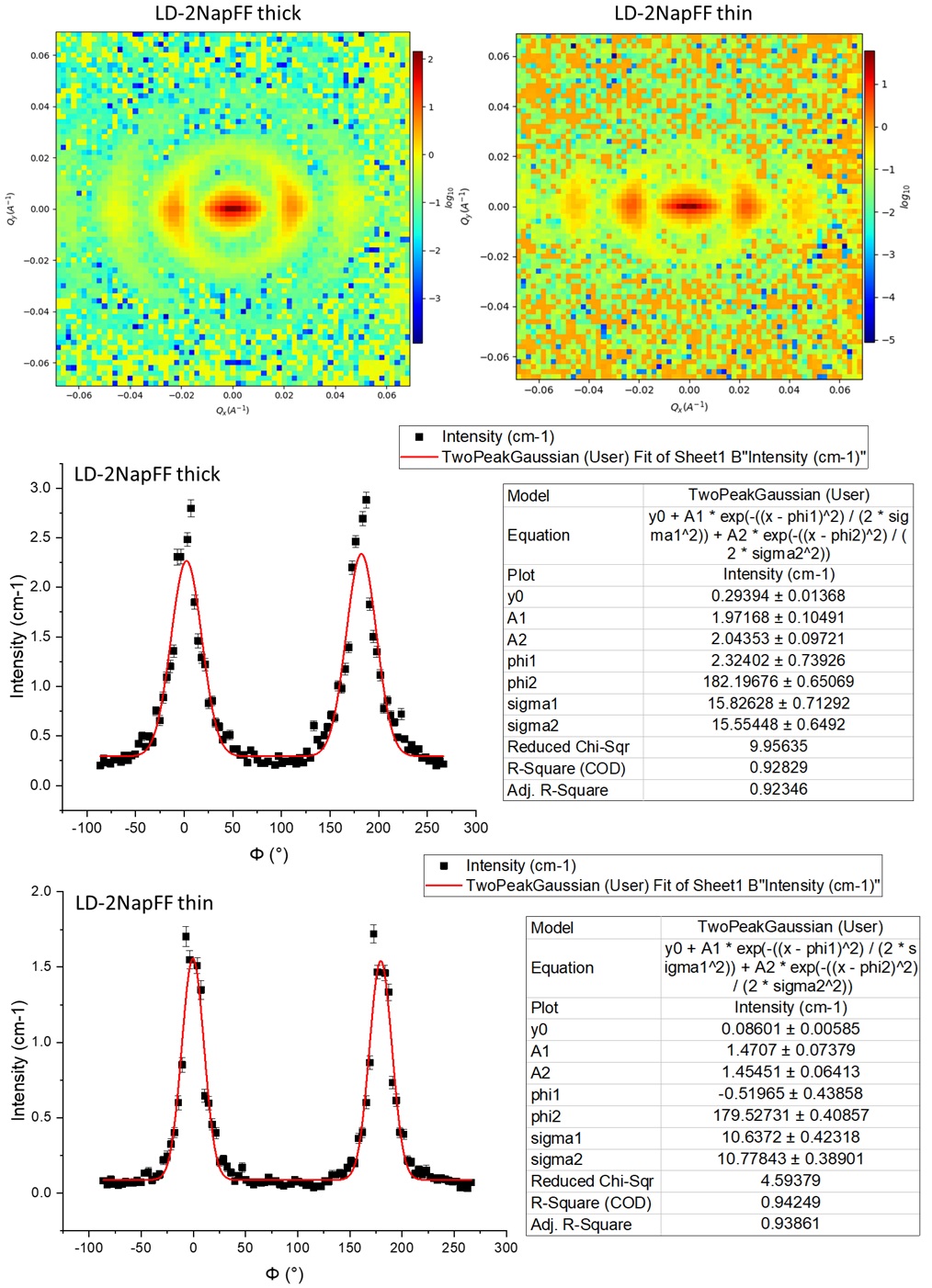


Figure S13: 2D SANS pattern of thick and thin LD-2NapFF noodles (top), and two peak Gaussian model fit of the azimuthal intensity (bottom).


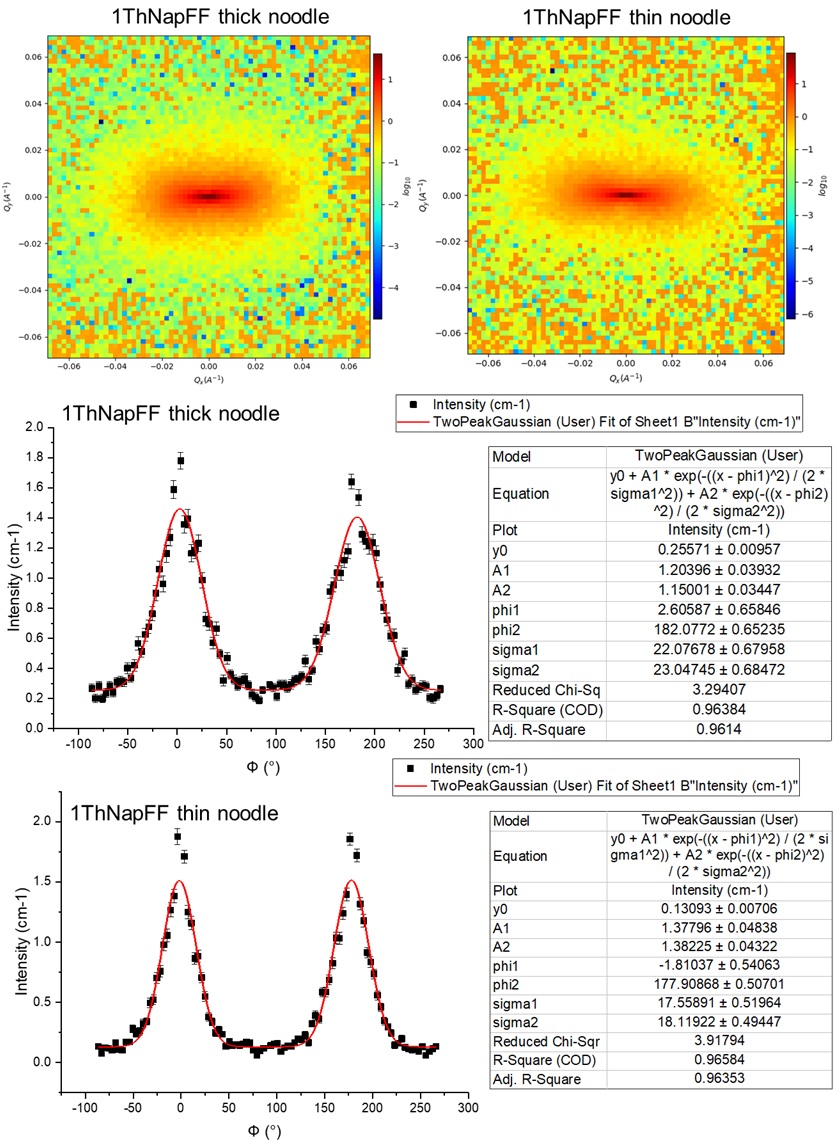


Figure S14: 2D SANS pattern of thick and thin 1ThNapFF noodles (top), and two peak Gaussian model fit of the azimuthal intensity (bottom).


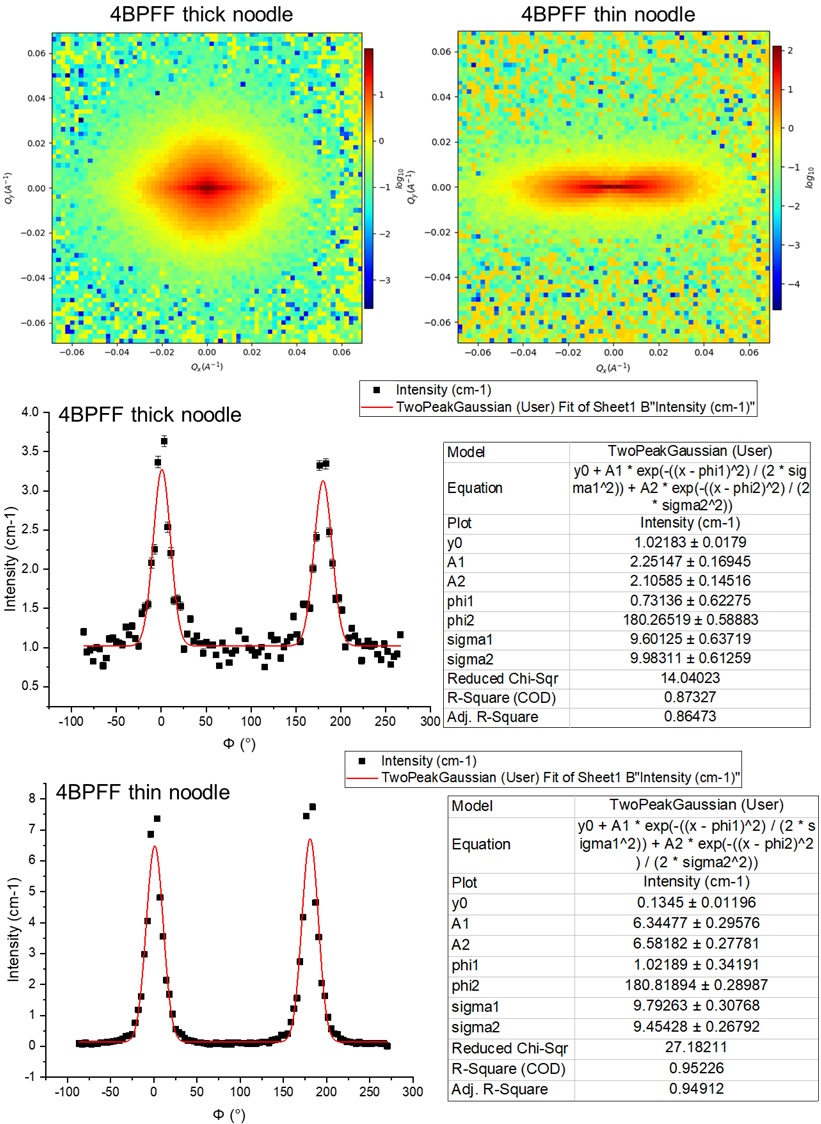


Figure S15: 2D SANS pattern of thick and thin 4BPFF noodles (top), and two peak Gaussian model fit of the azimuthal intensity (bottom).


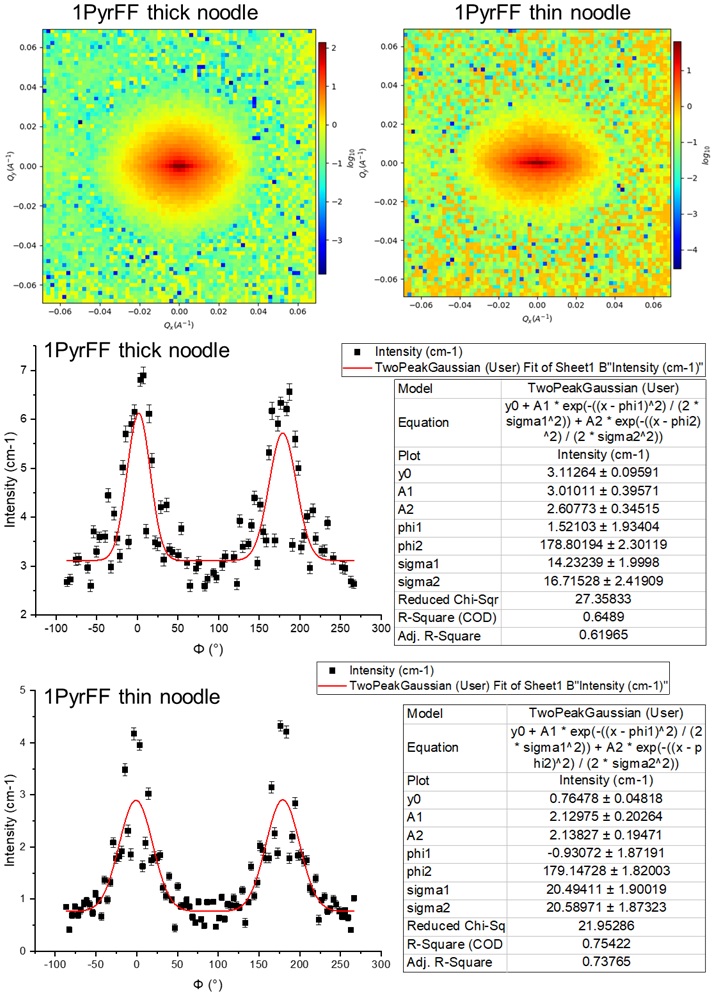


Figure S16: 2D SANS pattern of thick and thin 1PyrFF noodles (top), and two peak Gaussian model fit of the azimuthal intensity (bottom).


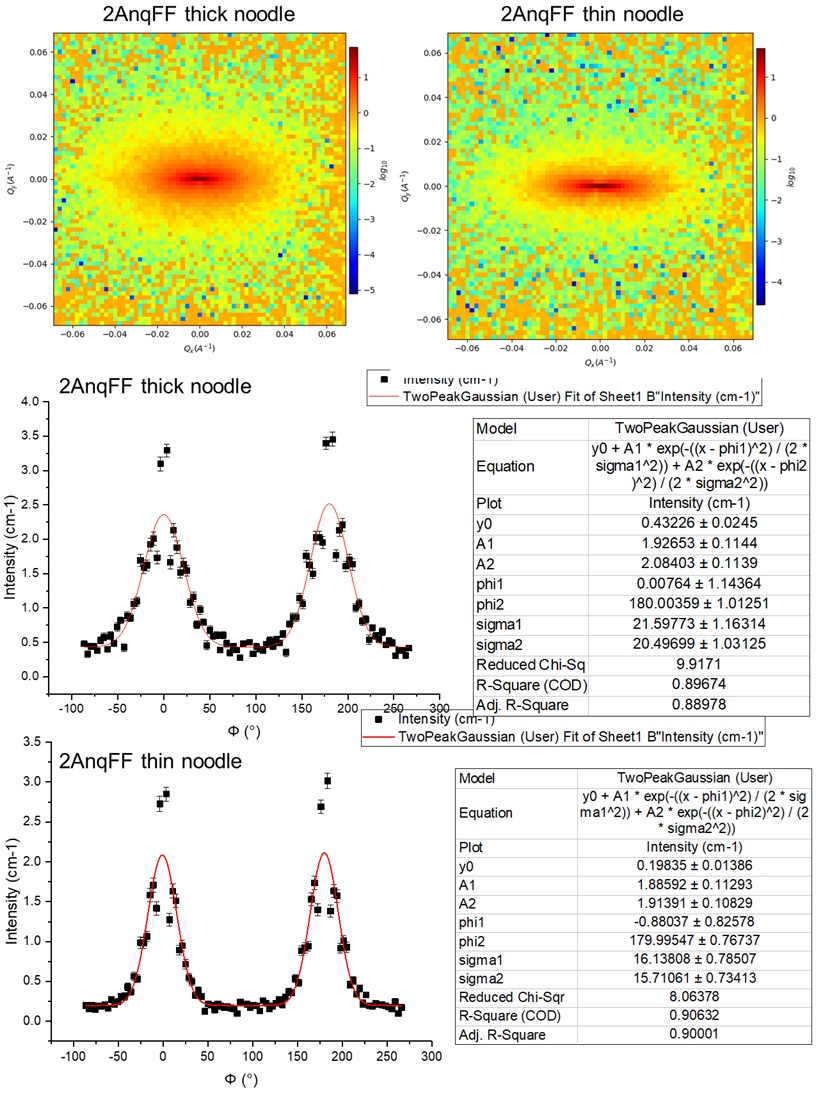


Figure S17: 2D SANS pattern of thick and thin 2AnqFF noodles (top), and two peak Gaussian model fit of the azimuthal intensity (bottom).

Calculating the Anisotropy A(q)

Quantification of fibrillar alignment from 2D SANS patterns using azimuthal intensity sector averaging has been previously employed to study shear-aligned micelles and peptide assemblies, often using Legendre polynomial fitting,^1^ or full azimuthal profiles to extract orientation parameters.^2^ To quantify the degree of fibrillar alignment in the gel noodles, we analyzed the 2D SANS patterns. Azimuthal sector averages were extracted from the scattering images along directions parallel and perpendicular to the long axis of the noodle. Specifically, I∥(q) denotes the scattering intensity along the direction of the noodle (aligned with the presumed fibrillar axis), and I⊥​(q) refers to the intensity perpendicular to this direction. These profiles were fitted using a two-peak Gaussian model (centered at ~0° and ~180° azimuthal angles), and the anisotropy parameter A(q) was calculated using a previously established formula:^3^

$\boldsymbol{A}\mathbf{(}\boldsymbol{q}\mathbf{)=}\frac{\boldsymbol{I}\boldsymbol{\|(}\boldsymbol{q}\boldsymbol{)} \mathbf{-}\boldsymbol{I}\mathbf{⏊(}\boldsymbol{q}\mathbf{)}}{\boldsymbol{I}\boldsymbol{\|}\left( \boldsymbol{q} \right)\mathbf{+}\boldsymbol{I}\mathbf{⏊(}\boldsymbol{q}\mathbf{)}}$ (Equation S1)

Here, I$\boldsymbol{\|}$(q) is the intensity along the direction of fibrilar alignment, composed of the baseline intensity (y_0_) plus the average peak height A_1_ and A_2_ (denoted as A_avg_).

I⏊(q) is the baseline intensity y_0_, representing scattering perpendicular to the alignment.

Thus,

$\boldsymbol{A}\mathbf{(}\boldsymbol{q}\mathbf{)=}\frac{\mathbf{A}_{\boldsymbol{avg}}\mathbf{+}\mathbf{y}_{\boldsymbol{0}}\mathbf{-}\mathbf{y}_{\boldsymbol{0}}}{\mathbf{A}_{\boldsymbol{avg}}\mathbf{+}\mathbf{y}_{\boldsymbol{0}}\mathbf{+}\mathbf{y}_{\boldsymbol{0}}}$ (Equation S2)

Or, $\boldsymbol{A}\mathbf{(}\boldsymbol{q}\mathbf{)=}\frac{\mathbf{A}_{\boldsymbol{avg}}}{\mathbf{A}_{\boldsymbol{avg}}\mathbf{+2}\mathbf{y}_{\boldsymbol{0}}}$  (Equation S3)

The associated error was calculated using the standard law of propagation of uncertainty (Gaussian error propagation)^4^:

$\boldsymbol{\sigma(}\boldsymbol{A}_{\boldsymbol{q}}\boldsymbol{)}\mathbf{=}\frac{\mathbf{2}}{{\mathbf{(}\mathbf{A}_{\boldsymbol{avg}}\mathbf{+2}\mathbf{y}_{\boldsymbol{0}}\mathbf{)}}^{\boldsymbol{2}}}\sqrt{\boldsymbol{y}_{\boldsymbol{0}}^{\boldsymbol{2}}\boldsymbol{\sigma}_{\boldsymbol{Aavg}}^{\boldsymbol{2}}\boldsymbol{+}\boldsymbol{A}_{\boldsymbol{avg}}^{\boldsymbol{2}}\boldsymbol{\sigma}_{\boldsymbol{y}\boldsymbol{0}}^{\boldsymbol{2}}}$ (Equation S4)

Where $\boldsymbol{\sigma}_{\boldsymbol{Aavg}}\mathbf{=}\frac{\sqrt{\boldsymbol{\sigma}_{\boldsymbol{A}\boldsymbol{1}}^{\boldsymbol{2}}\boldsymbol{+}\boldsymbol{\sigma}_{\boldsymbol{A}\boldsymbol{2}}^{\boldsymbol{2}}}}{\mathbf{2}}$ (Equation S5)

This formulation enables the quantification of directional scattering, with A(q) = 0 corresponding to isotropic scattering and A(q) = 1 indicating perfectly aligned structures.


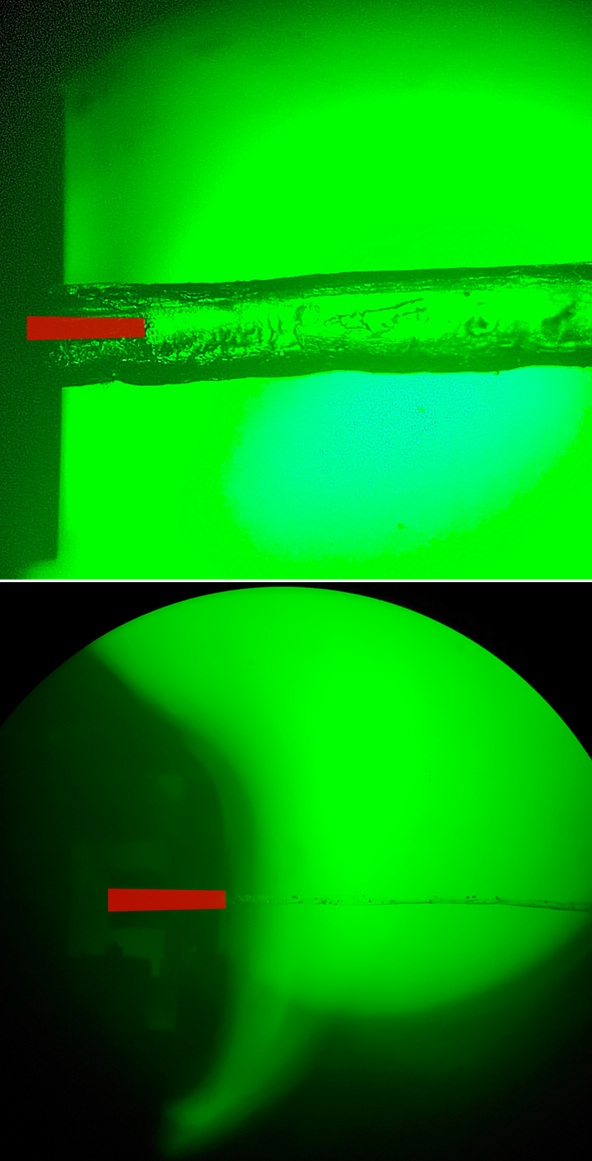


Figure S18: Microscope eyepiece view of 2NapFF thick and thin noodles mounted at the Chiaro nanoindenter. The cantilever attached to the probe is highlighted as a red box to visually represent the difference between the diameters of the two noodles.


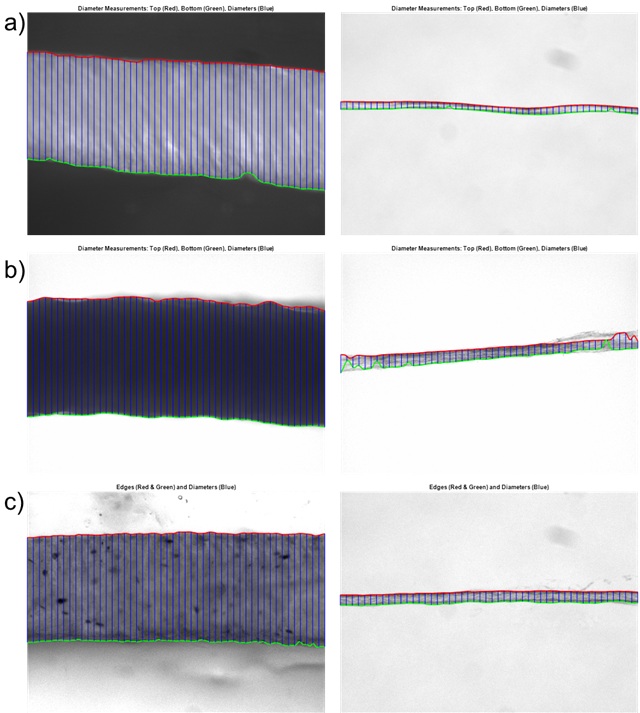


Figure S19: Evaluating the noodle diameters by canny edge detection in MATLAB: (a) 2NapFF, (b) LD-2NapFF, (c): 1ThNapFF.


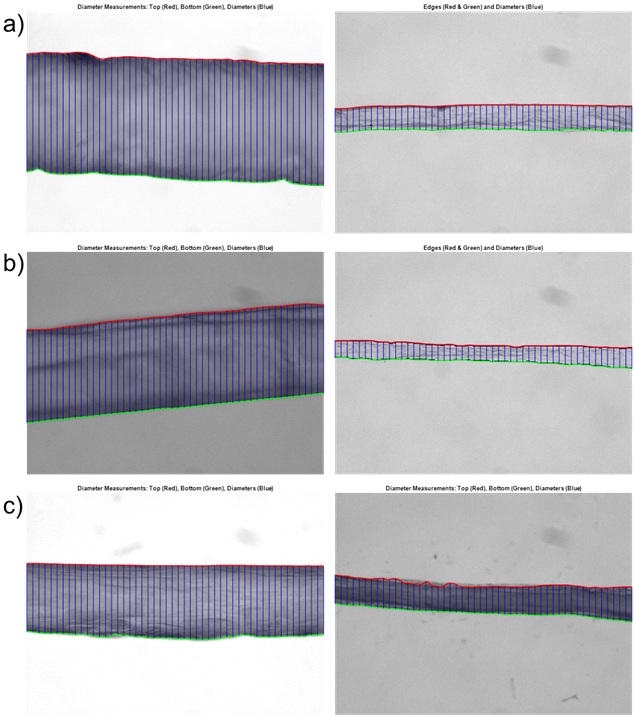


Figure S20: Evaluating the noodle diameters by canny edge detection in MATLAB: (a) 4BPFF, (b) 1PyrFF, (c): 2AnqFF.


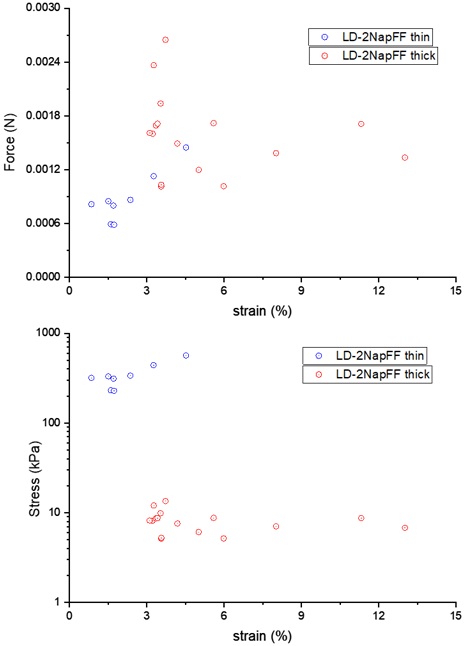


Figure S21. The force-strain (top) and stress-strain (bottom) profiles of the LD-2NapFF thick (red) and thin (blue) noodles.


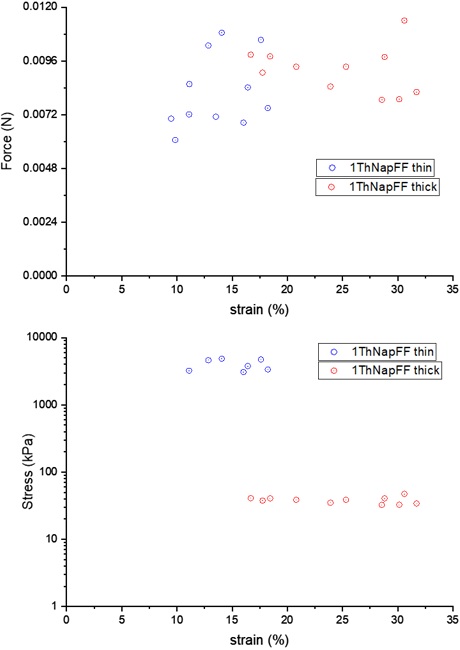


Figure S22. The force-strain (top) and stress-strain (bottom) profiles of the 1ThNapFF thick (red) and thin (blue) noodles.


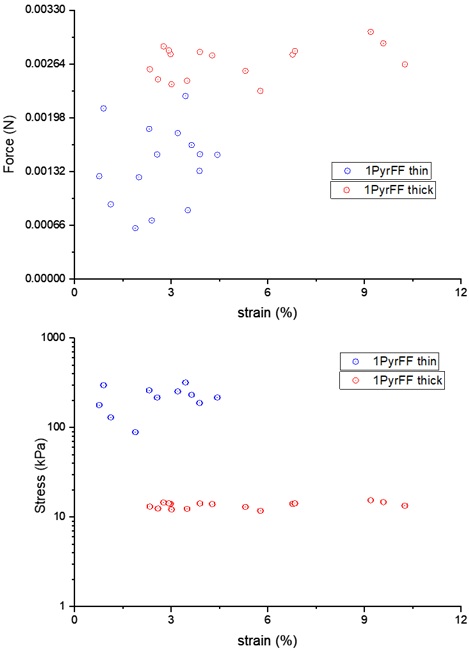


Figure S23. The force-strain (top) and stress-strain (bottom) profiles of the 1PyrFF thick (red) and thin (blue) noodles.


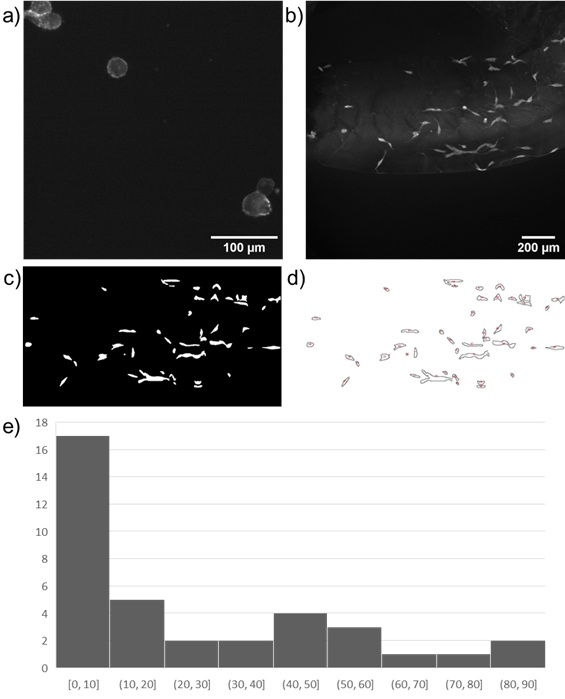


**Figure S24.** Quantitative analysis of C2C12 morphology and orientation on gel noodle segments with distinct processing histories. (a) Representative fluorescence image of C2C12 cells on the pump-on (thick) segment, which exhibits lower retained alignment. Cells are mostly round, so it is not possible to perform quantitative orientation analysis as near-circular cells do not have a well-defined major axis. (b) Representative image of C2C12 cells on the pump-off (thin) segment, which exhibits higher retained alignment and promotes elongated cell morphology. (c) The straightened region of interest from (b), generated in ImageJ (Version 1.54p) by tracing the noodle center line and applying the ‘straighten’ function, followed by thresholding to create a binary mask of cells. (d) Outlines of segmented cells identified by ImageJ using ‘Analyze Particles’, from which ellipse-fit parameters (major axis, minor axis, and angle) were extracted. (e) Relative orientation histogram (θrel) for elongated cells on the pump-off (thin) segment, where θrel is the angle between the ellipse-fit cell major axis and the noodle axis, wrapped to 0–90°. The distribution is clearly biased toward noodle axis, with ~46% of elongated cells oriented within 0–10° of the noodle axis.

References:

1. J. S. Weston, D. P. Seeman, D. L. Blair, P. F. Salipante, S. D. Hudson and K. M. Weigandt, *Rheologica Acta*, 2018, **57**, 241-250.

2. I. W. Hamley, S. Burholt, J. Hutchinson, V. Castelletto, E. R. da Silva, W. Alves, P. Gutfreund, L. Porcar, R. Dattani, D. Hermida-Merino, G. Newby, M. Reza, J. Ruokolainen and J. Stasiak, *Biomacromolecules*, 2017, **18**, 141-149.

3. E. R. Draper, M. Wallace, D. Honecker and D. J. Adams, *Chemical Communications*, 2018, **54**, 10977-10980.

4. P. R. Bevington and D. K. Robinson, *Data Reduction and Error Analysis for the Physical Sciences*, McGraw-Hill, 2003.
